# Combined Perioperative Lapatinib and Trastuzumab in Early HER2-Positive Breast Cancer Identifies Early Responders: Randomized UK EPHOS-B Trial Long-Term Results

**DOI:** 10.1158/1078-0432.CCR-21-3177

**Published:** 2022-02-14

**Authors:** Nigel Bundred, Nuria Porta, Adrian Murray Brunt, Angela Cramer, Andrew Hanby, Abeer M. Shaaban, Emad A. Rakha, Anne Armstrong, Ramsey I. Cutress, David Dodwell, Marie A. Emson, Abigail Evans, Sue M. Hartup, Kieran Horgan, Sarah E. Miller, Stuart A. McIntosh, James P. Morden, Jay Naik, Sankaran Narayanan, Jane Ooi, Anthony I. Skene, David A. Cameron, Judith M. Bliss

**Affiliations:** 1 Manchester University NHS Foundation Trust and University of Manchester, Manchester, United Kingdom.; 2 The Institute of Cancer Research, Clinical Trials and Statistics Unit, London, United Kingdom.; 3 University Hospitals of North Midlands and Keele University, United Kingdom.; 4 The Christie Pathology Partnership, Manchester, United Kingdom.; 5 Leeds Institute of Medical Research at St. James's, Leeds, United Kingdom.; 6 Queen Elizabeth Hospital Birmingham and University of Birmingham, Birmingham, United Kingdom.; 7 University of Nottingham, Nottingham, United Kingdom.; 8 The Christie NHS Foundation Trust, Manchester, United Kingdom.; 9 University of Southampton and University Hospital Southampton, Southampton, United Kingdom.; 10 Nuffield Department of Population Health, University of Oxford, Oxford, United Kingdom.; 11 Poole Hospital NHS Foundation Trust, United Kingdom.; 12 St James's University Hospital, Leeds, United Kingdom.; 13 Queen's University Belfast, Belfast, United Kingdom.; 14 Mid Yorkshire NHS Hospitals Trust, United Kingdom.; 15 University Hospitals of North Midlands, Stoke-on-Trent, United Kingdom.; 16 Royal Bolton Hospital, Manchester, United Kingdom.; 17 University of Southampton, Southampton, United Kingdom.; 18 University of Edinburgh Cancer Research Centre, Institute of Genetics and Cancer, Western General Hospital, Edinburgh, United Kingdom.

## Abstract

**Purpose:**

EPHOS-B aimed to determine whether perioperative anti-HER2 therapy inhibited proliferation and/or increased apoptosis in HER2-positive breast cancer.

**Patients and Methods:**

This randomized phase II, two-part, multicenter trial included newly diagnosed women with HER2-positive invasive breast cancer due to undergo surgery. Patients were randomized to: part 1 (1:2:2), no treatment (control), trastuzumab or lapatinib; part 2 (1:1:2) control, trastuzumab, or lapatinib and trastuzumab combination. Treatment was given for 11 days presurgery. Coprimary endpoints were change in Ki67 and apoptosis between baseline and surgery tumor samples (biologic response: ≥30% change). Central pathology review scored residual cancer burden (RCB). Relapse-free survival (RFS) explored long-term effects.

**Results:**

Between November 2010 and September 2015, 257 patients were randomized (part 1: control 22, trastuzumab 57, lapatinib 51; part 2: control 29, trastuzumab 32, combination 66). Ki67 response was evaluable for 223 patients: in part 1 Ki67 response occurred in 29/44 (66%) lapatinib versus 18/49 (37%) trastuzumab (*P* = 0.007) and 1/22 (5%) control (*P* < 0.0001); in part 2 in 36/49 (74%) combination versus 14/31 (45%) trastuzumab (*P* = 0.02) and 2/28 (7%) control (*P* < 0.0001). No significant increase in apoptosis after 11 days was seen in treatment groups. Six patients achieved complete pathologic response (pCR, RCB0) and 13 RCB1, all but two in the combination group. After 6 years median follow-up, 28 (11%) had recurrence and 19 (7%) died. No recurrences or deaths were observed among patients who achieved a pCR. Ki67% falls ≥50% associated with fewer recurrences (*P* = 0.002).

**Conclusions:**

Early response after short duration anti-HER2 dual therapy identifies cancers dependent on the HER2 pathway providing a strategy for exploring risk-adapted individualized treatment de-escalation.

**Translational Relevance**

In a randomized trial of 257 patients with HER2-positive breast cancer, lapatinib (alone 66% or in combination 74%) for 11 days produced higher Ki67 response rates than trastuzumab alone (37%–45%) or control (5%–7%). Combination treatment achieved a pCR or RCB1 in 26% cancers. After median follow-up of 6 years, perioperative falls in Ki67% of 50% or more were associated with a lower relapse rate than smaller or no decrease in Ki67. Early response to therapy identifies cancers dependent on the HER2 pathway, allowing individualization of treatment.

## Introduction

The human epidermal growth factor receptor 2 (HER2) is a tyrosine kinase receptor amplified or overexpressed in 15% to 20% of breast cancers. HER2 lacks a specific ligand, and signaling occurs after the formation of heterodimers with HER1 and HER3 (1). Targeting this pathway improves outcomes for patients with HER2-positive breast cancer. Trastuzumab interacts with the extracellular domain of the HER2 protein to inhibit its function (1, 2), but the mechanism of action is incompletely understood. Lapatinib blocks the HER1/2 internal tyrosine kinase domain and inhibits proliferation of HER2-positive cancers (3) as shown in a small preoperative trial (4).

Changes in proliferation biomarkers, including Ki67, predict clinical response and long-term outcome after 2 weeks of endocrine therapy in estrogen receptor (ER)-positive breast cancer (5–7). Incompletely excised breast cancers requiring re-excision within 48 days of surgery showed a significant increase in proliferation if they were HER2-positive, but not if they were HER2-negative (8). Preventing these early changes provides a rationale for window-of-opportunity studies investigating response to short-term treatment, enhancing prospects for personalizing medicine by identifying tumors sensitive to anti-HER2 therapy (without added chemotherapy).

The Effect of Perioperative Anti-HER2 therapy on Early Breast Cancer Study – Biological phase (EPHOS-B) was designed to assess whether either single-agent lapatinib or trastuzumab given as perioperative treatment had effects on Ki67 and/or apoptosis compared with no anti-HER2 therapy prior to surgery (part 1). Emerging evidence from the NeoSphere trial (9) on the safety and efficacy of combination anti-HER2 therapy led to a protocol amendment, enabling patient allocation between control, trastuzumab alone, or the combination of lapatinib and trastuzumab (part 2). Although the primary biological endpoint reported here is a short-term biomarker, its presentation is accompanied by analyses illustrating impact on 5-year disease outcomes and exploratory analysis associating response with stromal tumor-infiltrating lymphocytes (TILs).

## Patients and Methods

### Design and patients

EPHOS-B (NCT01104571) was a phase II, open-label, randomized, UK multicenter trial conducted in two parts, in newly diagnosed women with HER2-positive invasive breast cancer due to undergo surgery within 28 days. Patients had to be willing to undergo adjuvant chemotherapy and trastuzumab postsurgery as per standard of care and provide written informed consent for participation and donation of tissue and blood samples. Patients with significant cardiac abnormalities were ineligible. Baseline left ventricular ejection fraction (LVEF) ≥55% was required for trial entry. Full selection criteria are to be found in Appendix 1. The trial was conducted in accordance with Good Clinical Practice Guidelines and the Declaration of Helsinki.

### Procedures

In part 1, patients were randomized (1:2:2) to receive no perioperative treatment (control), trastuzumab alone, or lapatinib alone. In part 2, patients were allocated (1:1:2) to control, trastuzumab, or lapatinib and trastuzumab combined. Treatment commencement date was agreed on prior to randomization as 11 days (+2/−1) before the scheduled surgery. Treatment allocation was open-label, computer-generated, and centrally performed via telephone to the trials unit. In part 1, permuted blocks (up to size 12) stratified by center were used; in part 2, minimization with a random element and center as balancing factor was adopted to avoid imbalance given the smaller than expected number of patients randomized per center.

Trastuzumab (alone or in combination) was given intravenously before surgery on days 1 and 8 at an accelerated loading schedule dose of 6 mg/kg (to achieve faster steady-state levels of therapeutic efficacy; ref. 10) and one dose of 2 mg/kg was given after surgery between days 15 and 19. In part 1, lapatinib was given at a dose of 1,500 mg/day orally continuously for 28 days including the day of surgery. In part 2, when combined with trastuzumab, the lapatinib dose was 1,000 mg/day orally for 28 days.

Definitive surgery was according to local practice and patient choice. If nodal involvement was identified preoperatively, axillary clearance was the standard treatment. Adjuvant treatment was as per local policy and not influenced by EPHOS-B allocated treatment (see Appendix 1). Patients were followed up for cardiac toxicities and disease outcome every 6 months for 2 years after randomization, then annually.

### Assessment of biomarkers

Formalin-fixed, paraffin-embedded (FFPE) tumor blocks from diagnostic core biopsy and surgical specimens were centrally assessed for quality and tumor content, and analyzed for Ki67 and activated caspase 3 (apoptosis) by IHC using methods described previously (4, 5, 11). Hormone receptor status (ER and, when available, progesterone receptor [PgR]) was locally evaluated by IHC: Allred (or Quickscore), percentage tumor cells, or *H* score were recorded if available. The cut-off for positivity was ≥1% tumor cells, or Allred/Quickscore ≥3. HER2 was evaluated locally and judged positive by IHC 3+ score or FISH amplification (12, 13). FISH assessment was retrospectively repeated centrally. Central scoring of stromal TILs was conducted by specialist breast pathologists following international recommendations (14) on scanned H&E baseline and surgery slides.

Unexpectedly, a proportion of patients had insufficient tumor tissue in the surgical specimen for biomarker analysis. A review of pathology reports blinded to allocated treatment was undertaken to identify cases with evidence of potential tumor regression, and their pathology centrally assessed. Tumor bed sections at surgery were reviewed to confirm pathologic complete response (pCR) or, if the tumor were still present, to assess the two largest measurable spans of tumor, cellularity of the tumor within the tumor bed, and ductal carcinoma *in situ* (DCIS) within the tumor. Lymph node stage was recorded from pathology reports, and the size of the largest metastatic deposit measured. If there was detectable evidence of tumor response and nodal status was known, residual cancer burden (RCB) score and class [RCB0 (pCR); RCB1 (minimal residual disease); RCB2/3 (moderate or extensive residual disease)] were calculated (15). The remaining cases not selected for central pathology review were considered RCB2/3.

### Outcomes

The coprimary endpoints were changes in Ki67 and in apoptosis, with biological response defined as a relative decrease in Ki67 of >30%, or an increase in apoptosis of >30% between baseline and surgery (16). Secondary endpoints included relapse-free survival (RFS, time from randomization to local, regional, distant tumor recurrence, or death from any cause, with second primary cancers censored), overall survival, and safety. Exploratory endpoints included disease response at surgery, HER2 amplification by FISH, and changes in TILs during the perioperative period.

### Statistical analysis

The planned sample size (*N* = 250) assumed that biological response in Ki67 or apoptosis would be ≤5% in the control group compared with >30% in the treatment groups and powered to detect 30% differences in response rate between treatment groups. With 2.5% one-sided type I error, 85% power for the treatment-control comparisons, and >80% for between-treatment comparisons, 40 lapatinib (L, part 1), 55 control (C, part 1: 20, part 2: 35), 75 trastuzumab (T, part 1: 40, part 2: 35), and 80 combination (T + L, part 2) patients were required. Between-group comparisons were restricted to concurrently randomized patients: L versus C (part 1), L versus T (part 1), T + L versus T (part 2), T + L versus T (part 2), and T versus C (part 1 and part 2).

Perioperative change endpoints were analyzed on all patients who had paired biological data; patients who were found ineligible before starting any treatment were excluded. Surgical Ki67 and apoptosis scores in patients with breast pCR (regardless of nodal status) were excluded from the analysis. We conducted a sensitivity analysis on Ki67% assuming such patients had a 0% score at surgery. Percentage changes by randomized treatment group were compared by Mann–Whitney tests. The proportion of patients responding in Ki67 and/or apoptosis analyses were compared using Fisher exact test. An alternative threshold of >50% reduction (as used in the MAPLE trial; ref. 4) was also explored. The proportion of patients with pCR or RCB1 was described for each treatment group.

All randomized patients were included in the analysis of time-to-event endpoints, summarized by Kaplan–Meier estimates, and groups compared with log-rank tests. Association of perioperative biological changes with RFS was considered exploratory in nature; part 1 and part 2 were combined for this, and log-rank tests stratified by treatment group. Perioperative %Ki67 decrease was categorized into decrease of 50% or more, 10% to 50% decrease; or no relevant decrease (<10% decrease or no decrease). Absolute Ki67 values were categorized following on from work in endocrine sensitive breast cancer (7): baseline and surgery Ki67 were high if ≥10% or low if <10%, and combined into “high–high,” “high–low,” “low–high,” or “low–low” categories. Patients with pCR or 0% breast cellularity were imputed a value of 0% at surgery and included in these analyses.

TILs were measured as a percentage (occupation of TILs in the tumor stromal surface area) and categorized into low (≤20%) or high TILs (>20%; ref. 17). Analysis of changes in TILs was restricted to patients with paired baseline and surgery data and no evidence of tumor regression at surgery (i.e., RCB2/3) to account for the lack of samples to perform analyses in pCR and patients in the RCB1 group. TILs were associated with trial outcomes, for which part 1 and part 2 data were combined.

A 5% significance level was considered for treatment comparisons of primary and secondary endpoints and 1% for all other exploratory analyses. Stata (v13.0 or later) statistical software was used. Data cut-off for biomarker analyses was July 14, 2017; updated for 5-year outcomes on December 20, 2020. Further details of the methodology are available in the Appendix 1.

### Data availability

Formal requests for sharing the data generated in this study will be considered with due regard given to funder and sponsor guidelines. Requests involving collaboration with the EPHOS-B Trial Management Group (TMG) are strongly encouraged. Requests are reviewed by the TMG and will be considered dependent on scientific merit, ethical considerations including patient consent, funding, resources, and alignment with the trial objectives. Data sharing are further approved by the Trial Steering Committee.

## Results

### Patient disposition and baseline characteristics

**Figure 1.**
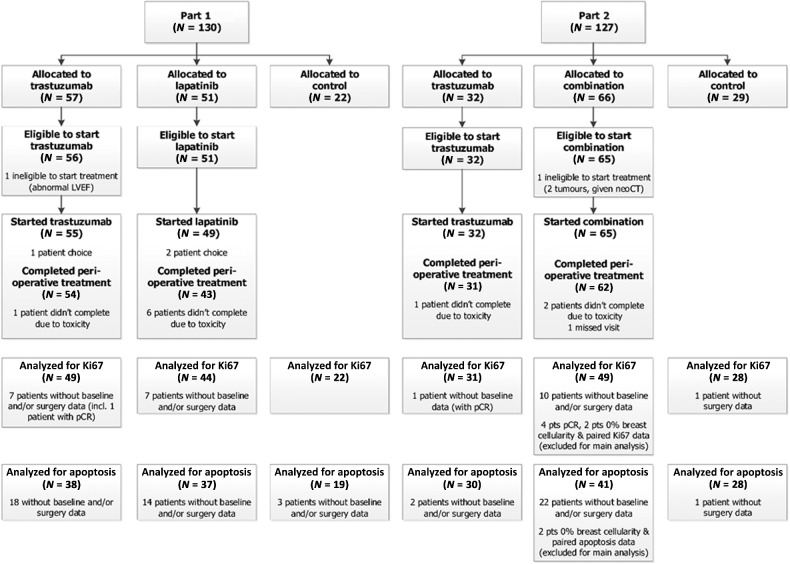
The CONSORT diagram summarizes patients recruited into each part of the trial, patients randomized, patients eligible to start treatment, patients who started treatment, and those who completed perioperative treatment as per protocol. In part 1, 22 patients were allocated to control, 57 to trastuzumab, and 51 to lapatinib; in part 2, 29 were allocated to control, 32 to trastuzumab, and 66 to the combination. Overall, 255 (99%) patients were considered eligible to start treatment and included in the analysis of perioperative endpoints. Of the 204 patients in the treatment groups, 201 patients (99%) received some perioperative treatment, with 190/201 (95%) completing the 11 days of perioperative treatment. The figure also describes how many patients available for analysis of coprimary endpoints Ki67 and apoptosis. Only patients with both paired samples and enough tumor tissue for biomarker analysis were included in the analysis: 223 patients (88%) had paired Ki67 data and 193 (76%) had paired apoptosis data available for analysis. Patients with pCR or 0% breast cellularity were excluded from main analysis of Ki67 and apoptosis.

Two hundred fifty-seven patients were recruited from 21 UK centers; 130 entered part 1 between November 15, 2010, and July 29, 2013, and 127 entered part 2 between August 6, 2013 and September 10, 2015. Two patients (1%) were found ineligible before starting treatment and excluded from the analysis of perioperative endpoints (Fig. 1). Overall, 172 patients (67%) had ER-positive tumors, with a median tumor size of 2.2 cm (Table 1). Details of adjuvant treatment following surgery are provided in Appendix 2; with no differences between randomized groups in adjuvant treatment received.

**Table 1.** Patient demographics and tumor characteristics at baseline and at surgery, by randomized treatment group.

	PART 1						PART 2							
	Trastuzumab		Lapatinib		Control		Trastuzumab		Combination		Control		Total	
	*N* = 57		*N* = 51		*N* = 22		*N* = 32		*N* = 66		*N* = 29		*N* = 257	
	No.	%	No.	%	No.	%	No.	%	No.	%	No.	%	No.	%
Patient demographics														
Age (y)														
Median (IQR)	50 (47–62)		51 (48–60)		53 (50–62)		52 (48–55)		53 (47–63)		58 (49–66)		53 (48–62)	
Menopausal status^a^														
Premenopausal	24	42	21	41	4	18	11	34	25	38	9	31	94	37
Peri-/postmenopausal	33	58	30	59	18	82	21	66	41	62	20	69	163	63
Tumor characteristics from the diagnostic core														
Grade														
Grade 1	3	5	0	0	0	0	0	0	2	3	0	0	5	2
Grade 2	20	35	21	41	9	41	14	44	26	39	13	45	103	40
Grade 3	28	49	24	47	13	59	17	53	36	55	14	48	132	51
Unknown^b^	6	11	6	12	0	0	1	3	2	3	2	7	17	7
Histology type														
Infiltrating ductal (no special type)	54	95	45	88	22	100	29	91	59	89	27	93	236	92
Infiltrating lobular	0	0	3	6	0	0	2	6	4	6	1	3	10	4
Mixed	0	0	2	4	0	0	1	3	0	0	1	3	4	2
Mucinous	1	2	1	2	0	0	0	0	1	2	0	0	3	1
Infiltrating micropapillary	0	0	0	0	0	0	0	0	1	2	0	0	1	0
Not known	2	4	0	0	0	0	0	0	1	2	0	0	3	1
Tumor size (cm)^c^														
<2	26	46	21	41	11	50	19	59	39	59	18	62	134	52
2–5	25	44	27	53	10	46	13	41	26	39	11	38	112	44
≥5	6	11	3	6	1	5	0	0	1	2	0	0	11	4
HER2 locally assessed by														
IHC (IHC 3+)	53	93	46	90	17	77	30	94	61	92	28	97	235	91
FISH (IHC 2+ confirmed by FISH)	4	7	5	10	5	23	2	6	5	8	1	3	22	9
HER2 centrally assessed (FISH)^d^														
HER2 amplified	52	91	46	90	21	95	29	91	58	88	29	100	235	91
HER2 not amplified	4	7	4	8	1	5	3	9	1	2	0	0	13	5
FISH data not available	1	2	1	2	0	0	0	0	7	10	0	0	9	4
ER (local assessment)														
Negative	20	35	20	39	7	32	11	34	15	23	12	41	85	33
Positive	37	65	31	61	15	68	21	66	51	77	17	59	172	67
PgR (local assessment)														
Negative	20	35	23	45	8	36	16	50	28	42	17	59	112	44
Positive	21	37	17	33	6	27	8	25	18	27	5	17	75	29
Missing	16	28	11	22	8	36	8	25	20	30	7	24	70	27
Details of surgery														
Definitive breast surgery														
Conservative surgery	26	46	22	43	14	64	20	63	45	68	18	62	145	56
Mastectomy	31	54	29	57	8	36	12	37	21	32	11	38	112	44
Definitive axillary surgery^e^														
Yes	57	100	51	100	22	100	32	100	66	100	28	97	256	99.6
Axillary node clearance	23	41	25	49	6	27	10	31	16	24	9	31	89	34.6
Level 1 sampling	3	5	1	2	0	0	1	3	5	8	2	7	12	4.7
Sentinel lymph node biopsy	31	54	25	49	16	73	21	66	45	68	17	59	155	60.3
No	0	0	0	0	0	0	0	0	0	0	1	3	1	0
Tumor features at surgery														
No. of lymph nodes involved														
0	35	61.4	25	49.0	16	72.7	19	59.4	48	72.7	19	65.5	162	63.0
1–3	14	24.6	17	33.4	4	18.2	8	25.0	12	18.2	7	24.1	62	24.1
4–9	6	10.5	7	13.7	2	9.1	1	3.1	5	7.6	2	6.9	23	9.0
10+	2	3.5	2	3.9	0	0.0	4	12.5	1	1.5	1	3.5	10	3.9
No. of lymph nodes examined														
1–3	29	50.9	23	45.1	12	54.6	19	59.4	45	68.2	14	48.3	142	55.2
4–9	10	17.5	6	11.8	5	22.7	4	12.5	8	12.1	6	20.7	39	15.2
≥10	18	31.6	22	43.1	5	22.7	9	28.1	13	19.7	9	31.0	76	29.6
Grade														
Grade 1	2	3.5	3	5.9	1	4.5	1	3.1	2	3.0	0	0.0	9	3.5
Grade 2	15	26.3	21	41.1	2	9.1	8	25.0	23	34.9	5	17.2	74	28.8
Grade 3	39	68.4	26	51.0	19	86.4	22	68.8	28	42.4	24	82.8	158	61.5
GX	0	0.0	1	2.0	0	0.0	0	0.0	0	0.0	0	0.0	1	0.4
Not known	1	1.8	0	0.0	0	0.0	1	3.1	13	19.7	0	0.0	15	5.8
Tumor size (cm)														
<2	26	45.6	18	35.3	9	40.9	18	56.2	43	65.2	14	48.3	128	49.8
2–5	26	45.6	30	58.8	12	54.6	11	34.4	22	33.3	15	51.7	116	45.1
≥5	5	8.8	3	5.9	1	4.5	3	9.4	0	0.0	0	0.0	12	4.7
Missing	0	0.0	0	0.0	0	0.0	0	0.0	1	1.5	0	0.0	1	0.4

Abbreviations: ER, estrogen receptor; FISH, fluorescence in situ hybridization; HER2, human epidermal growth factor receptor 2; IHC, immunohistochemistry; IQR, interquartile range; PgR, progesterone receptor.

^a^Includes nine patients (two trastuzumab part 2; five combination part 2; two control part 2) with missing menopausal status data who have been classified on the basis of their age (<50 = premenopausal, ≥50 = peri-/postmenopausal).

^b^Some UK hospitals do not routinely report grade on the diagnostic core.

^c^Presurgery, this measurement is either by ultrasound or clinical examination.

^d^For patients with local FISH testing, scores extracted from pathology reports.

^e^Percentage of mastectomy (*P* = 0.017) and axillary clearance (*P* = 0.047) were found to be lower in part 2 than part 1.

### Disease response

Forty of 255 patients showed evidence of potential tumor regression in the central review of pathology reports and underwent central RCB scoring; the remainder were considered RCB2/3. Although this analysis was originally unplanned, it became an essential component of the main trial analysis to inform impact of disease regression on the primary biomarker endpoints (informative censoring).

In part 1, 1/56 (2%) pCR was observed in the trastuzumab group. In part 2, 1/32 (3%) pCR occurred in the trastuzumab group, whereas in the combination group 4/65 (6%) achieved pCR, and 13/65 (20%) RCB1 were identified, including two-node positive patients who were node negative at surgery (Appendix 3). Two further combination treated patients who had scored RCB1 and RCB2/3 (due to nodal involvement) showed no residual disease in the breast (0% breast cellularity). Among the 19 patients in the pCR or RCB1 group, 14 (74%) had ER-positive tumors (Appendix 3). All but one (patient choice) received adjuvant chemotherapy as per local practice.

Before therapy, median (min–max) radiologic tumor size was 2 cm (0.9–2.8) for patients who achieved pCR, 1.4 cm (0.5–4.5) for RCB1 patients, and 1.9 cm (0.1–10) for RCB2/3 patients. In a multivariable analysis in the combination group, only size of tumor was associated with observing pCR/RCB1 response (Appendix 3).

### Ki67

**Figure 2.**
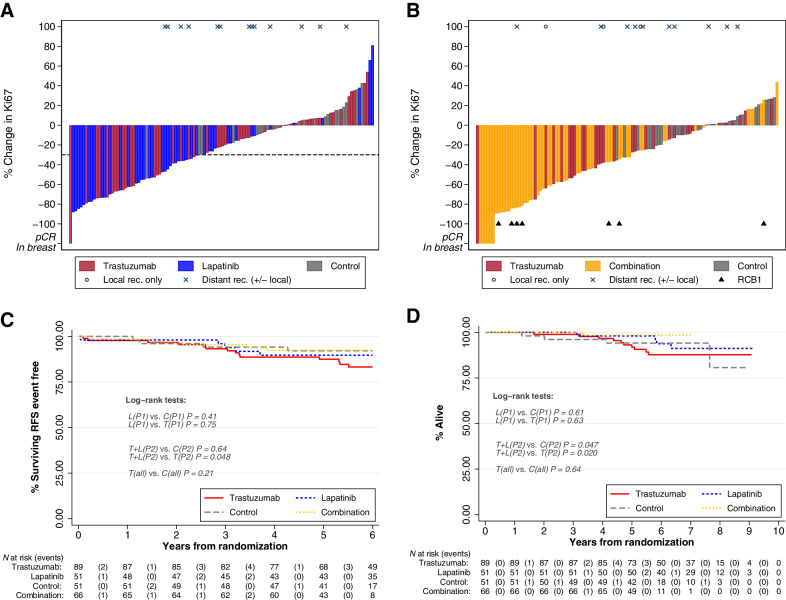
Percentage change in Ki67 between pretreatment (baseline) and surgery for part 1 (**A**) and part 2 (**B**); Kaplan–Meier estimates by treatment group for relapse free survival (**C**) and overall survival (**D**). **A,** Waterfall plots for part 1 and part 2: for each patient, bar height represents percentage change at surgery from baseline. Percentage change was calculated as [(surgery score + 0.1) − (pretreatment score + 0.1)]/[(pretreatment score + 0.1)]*100. The constant of 0.1 was added to accommodate cases with a value of 0%. Negative values represent decrease from baseline, positive values represent increase from baseline. pCR in breast: patients with pCR (no disease in ether breast or nodes) plus two additional patients with 0% breast cellularity but nodal involvement are represented as bars of height −120% at the left of the figures and noted “pCR in breast;” any existing Ki67 values for these patients have been excluded of the main analysis; in a sensitivity analysis, we imputed a value of −100% change for these patients (Appendix 2). Small triangles indicate patients with RCB1. Disease recurrences are also indicated at the top of each figure with circles and crosses. **B,** RFS is represented in the time interval of up to 6 years after randomization, as no RFS event occurred later. Overall survival is represented in the fully observed range of values. Log-rank test comparing concurrently randomized treatment groups are reported in the figures. In the figure, trastuzumab and control part 1 and part 2 groups are combined to improve readability. C, control; L, lapatinib; T, trastuzumab; T+L, combination; P1, part 1; P2, part 2; all, P1&P2; *P*, *P* value.

Waterfall plots illustrating the range of percentage change in Ki67 observed in individual patients are presented in Fig. 2A and B.

In part 1, 29/44 (66%) patients taking lapatinib had a Ki67 response (≥30% reduction) compared with 18/49 (37%) patients taking trastuzumab (*P*_LvT_ = 0.007) and 1/22 (5%) patients in the control group. (*P*_LvC_ < 0.0001). Median percentage change in Ki67 was −43% (IQR, −68% to −21%) with lapatinib, −14% (IQR, −51% to +6%) with trastuzumab, and +2% (IQR, −9% to +15%) with control (Table 2, Appendix 4.1).

**Table 2.** Analysis of Ki67 and apoptosis, by randomized treatment group.

	PART 1			PART 2		
	Trastuzumab	Lapatinib	Control	Trastuzumab	Combination	Control
Ki67						
*N* with paired data	49	44	22	31	49	28
Baseline Ki67 - median (IQR)	35 (28 to 45)	34 (27 to 42)	37 (24 to 49)	45 (37 to 57)	40 (29 to 54)	42 (31 to 54)
Surgery Ki67 - median (IQR)	31 (18 to 40)	20 (10 to 30)	35 (25 to 54)	30 (22 to 47)	20 (9 to 34)	39 (29 to 48)
Percentage change - median (IQR)	−14 (−51 to 6)	−43 (−68 to −21)	2 (−9 to 15)	−26 (−46 to −6)	−49 (−78 to −25)	−2 (−20 to 7)
Log-fold change - median (IQR)	−0.2 (−0.7 to 0.1)	−0.6 (−1.2 to −0.2)	0.0 (−0.1 to 0.1)	−0.3 (−0.6 to −0.1)	−0.7 (−1.5 to −0.3)	0.0 (−0.2 to 0.1)
Mann–Whitney test for % change – control vs. lapatinib/combination		*P* < 0.0001			*P* < 0.0001	
Mann–Whitney test for % change – trastuzumab vs. lapatinib/combination	*P* = 0.0034			*P* = 0.0054		
Response (Ki67 decrease >30%) – *N* (%)	18 (36.7%)	29 (65.9%)	1 (4.5%)	14 (45.2%)	36 (73.5%)	2 (7.1%)
95% CI	[23.4% to 51.7%]	[50.1% to 79.5%]	[0.1% to 22.8%]	[27.3% to 64.0%]	[58.9% to 85.1%]	[0.9% to 23.5%]
Fisher exact test – control vs. lapatinib/combination		*P* < 0.0001			*P* < 0.0001	
Fisher exact test – trastuzumab vs. lapatinib/combination	*P* = 0.007			*P* = 0.02		
Apoptosis						
*N* with paired data	38	37	19	30	41	28
Baseline apoptosis - median (IQR)	7 (5 to 9)	7 (5 to 9)	7 (6 to 9)	5 (3 to 8)	7 (5 to 9)	7 (4 to 10)
Surgery apoptosis - median (IQR)	8 (4 to 10)	4 (3 to 7)	8 (7 to 13)	6 (3 to 8)	3 (2 to 10)	7 (5 to 12)
Percentage change - median (IQR)	−5 (−18 to 21)	−25 (−42 to 1)	24 (−10 to 57)	4 (−32 to 48)	−34 (−56 to 10)	−2 (−15 to 63)
Log-fold change - median (IQR)	−0.1 (−0.2 to 0.2)	−0.3 (−0.5 to 0.0)	0.2 (−0.1 to 0.5)	0.0 (−0.4 to 0.4)	−0.4 (−0.8 to 0.1)	0.0 (−0.2 to 0.5)
Mann–Whitney test for % change – control vs. lapatinib/combination		*P* = 0.0002			*P* = 0.004	
Mann–Whitney test for % change – trastuzumab vs. lapatinib/combination	*P* = 0.01			*P* = 0.03		
Response (apoptosis increase >30%) – *N* (%)	7 (18.4%)	2 (5.4%)	7 (36.8%)	11 (36.7%)	8 (19.5%)	10 (35.7%)
95% CI	[7.7% to 34.3%]	[0.7% to 18.2%]	[16.3% to 61.6%]	[19.9% to 56.1%]	[8.8% to 34.9%]	[18.6% to 55.9%]
Fisher exact test – control vs. lapatinib/combination		*P* = 0.01			*P* = 0.17	
Fisher exact test – trastuzumab vs. lapatinib/combination	*P* = 0.15			*P* = 0.17		

Note: Table summarizes Ki67 and apoptosis at baseline (pretreatment), at surgery and the changes baseline-surgery. Change is summarized by percentage change = [(surgery score + 0.1) − (baseline score + 0.1)] × 100/(baseline + 0.1) and log-fold change = log[(surgery score + 0.1)/(baseline score + 0.1)]. Negative values indicate decrease from baseline; positive values indicate increase from baseline.

In part 2, 36/49 (74%) patients in the combination group had a Ki67 response compared with 14/31 (45%) patients in the trastuzumab group (*P*_T+LvT_ = 0.02) and 2/28 (7%) patients in the control group (*P*_T+LvC_ < 0.0001). Median percentage change in Ki67 was −49% (IQR, −78% to −25%) with combination, −26% (IQR, −46% to −6%) with trastuzumab and −2% (IQR, −20% to +7%) with control (Table 2, Appendix 4.1).

When combining part 1 and part 2, 32/80 (40%) patients in the trastuzumab group had a Ki67 response compared with 3/50 (6%) patients in the control group (*P*_TvC_ < 0.001). Median percentage change in Ki67 was −20% (IQR, −50% to +2%) with the trastuzumab group and 0% (IQR, −13% to +11%) in the control group.

In sensitivity analyses where 0% Ki67 at surgery was imputed in patients with breast pCR, similar results were obtained (Appendix 4.2). Treatment differences remained after adjusting for known prognostic factors (Appendix 4.3). In exploratory multivariable analyses of the pooled dataset, no other factors (including ER and PgR status) were found to be associated with Ki67 decrease (Appendix 4.4).

*HER2* gene amplification ratio (HER2/CEP17 ratio) by FISH (centrally assessed) correlated with change in Ki67 in the trastuzumab group, both in part 2 (*P* = 0.008), and part 1 and part 2 combined (*P* = 0.04); no association was found between amplification ratio and other trial outcomes (Appendix 5).

### Apoptosis

In part 1, 2/37 (5%) patients in the lapatinib group had an apoptosis response (>30% increase) compared with 7/38 (18%) patients in the trastuzumab group (*P*_LvT_ = 0.15) and 7/19 (37%) patients in the control group (*P*_LvC_ = 0.01). Median percentage change in apoptosis was −25% (IQR, −42% to +1%) with lapatinib, −5% (IQR, −18% to +21%) with trastuzumab, and +24% (IQR, −10% to +57%) with control (Table 2).

In part 2, 8/41 (20%) patients in the combination group had an apoptosis response compared with 11/30 (37%) in the trastuzumab group (*P*_T+LvT_ = 0.17) and 10/28 (36%) in the control group (*P*_T+LvC_ = 0.17). Median percentage change in apoptosis was −34% (IQR, −56% to +10%) with combination, +4% (IQR, −32% to +48%) with trastuzumab, and −2% (IQR, −15% to +63%) with control (Table 2).

When combining parts 1 and 2, 18/68 (26%) patients in the trastuzumab group had an apoptosis response compared with 17/47 (36%) patients in the control group (*P*_TvC_ = 0.31). Median percentage change in apoptosis was +2% (IQR, −28% to +36%) with trastuzumab and +6% (IQR, −12% to +62%) with control.

Changes from baseline in apoptosis correlated positively with changes in proliferation only in the combination group (*P* = 0.034; Appendix 6).

### Five-year time-to-event outcomes

After median follow-up of 6 years (IQR, 5.2–7.4), 28 women (11%) had breast cancer recurrence and 19 patients died, with all but one due to breast cancer following recurrence (Appendix 7). The proportion free from breast cancer recurrence at 5 years (5yr-RFS; 95% CI) was, in part 1, trastuzumab 88% (76–94), lapatinib 90% (77–96), and control 95% (72–99); in part 2, trastuzumab 87% (69–95), combination 92% (83–97), and control 90% (71–97; Fig. 2B). When combining part 1 and part 2, 5yr-RFS were trastuzumab 87% (79–93) and control 92% (80–97). There were no significant differences between randomized groups (Fig. 2C), even when adjusting by known prognostic factors (Appendix 7.1), although the study was not powered for such comparisons. Overall survival is shown in Fig. 2D. None of the patients with pCR recurred or died; only one patient in the RCB1 group had an RFS event (local recurrence).

**Figure 3.**
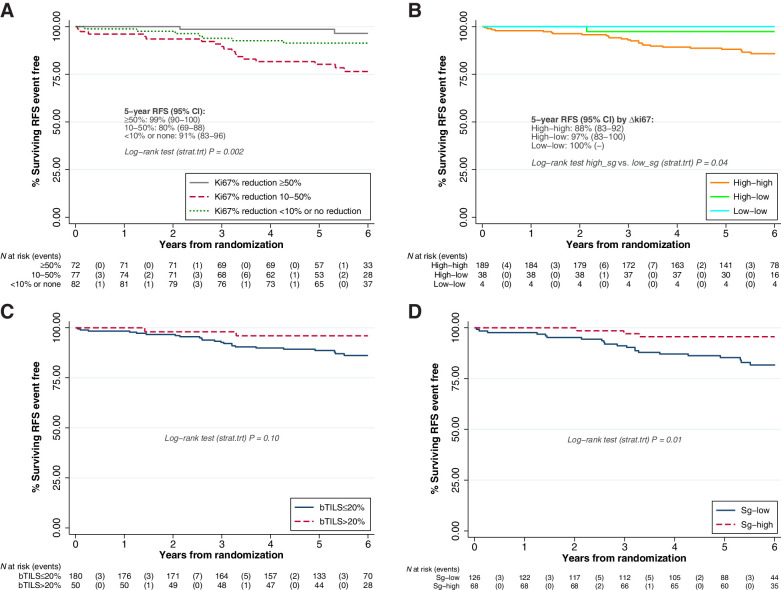
Association of perioperative changes in biological markers with RFS (**A**) by categories of Ki67 relative change, (**B**) by categories of Ki67 absolute change, (**C**) by baseline TILs, (**D**) by surgery TILs. RFS is represented in the time interval 0 to 6 years, as no RFS events occurred beyond 6 years from randomization. All treatment groups are combined; log-rank tests are stratified by treatment group (*P* = *P* value). For **A** and **B**, a value of −100% Ki67 change (ΔKi67) has been imputed for patients with a pCR in breast. For **B**, we have categorized both baseline and surgery Ki67 into high if ≥10% or low if <10%. No patient increased Ki67 from low to high after 11 days of perioperative treatment. Because of small number of patients in the “low–low” group, we have compared patients with “high” value at surgery with patients with “low” value at surgery.

For analysis of perioperative Ki67 changes and RFS, 231/257 patients were included. When categorizing Ki67 change (Fig. 3A), 2/72 (2.8%) RFS events (local only recurrences, one of these followed by distant recurrence) were observed in the group with Ki67 reductions ≥50%, whereas 17/77 (22%) RFS events (15 distant recurrences, two local only) occurred in the group with reductions between 10% and 50%, and 7/82 (8.5%) RFS events (six distant, one local only) were observed in the group with no relevant reduction; RFS was significantly different between the three groups (*P* = 0.002). Such differences remain in multivariable analysis with other prognostic factors (Appendix 7.2). When categorizing absolute Ki67 values, 189 patients (82%) remained with Ki67 high after 11 days of perioperative treatment (“high–high”), 38 patients (16%) reduced to low (“high–low”), and 4 (1.7%) remained low (“low–low”). No patient increased Ki67 from low to high after 11 days of perioperative treatment. Of the 26 RFS events observed, all but one (the local recurrence in a patient with RCB1 response) occurred in the “high–high” group (Fig. 3B).

### Exploratory analyses on stromal TILs

Baseline TILs (bTILs) could be scored for 230/255 patients (90%); 50 carcinomas (22%) showed high bTILs (>20%); no significant differences were found in bTILs between randomized groups (Appendix 8). We did not observe an association between bTILs and disease response (*P* = 0.58). When associated with RFS (Fig. 3C), 2/50 (4%) high bTILs experienced an RFS event, versus 23/180 (13%) among patients with low bTILs (*P* = 0.06).

Ki67 change was −33% (IQR, −62 to −8) for carcinomas with high bTILs and −23% (IQR, −56 to 2) for low bTILs (*P* = 0.19). In the trastuzumab group, Ki67 responses were observed in 8/13 (62%) high bTILs versus 23/62 (37%) low bTILs (*P* = 0.10, Appendix 8).

Change from baseline TILs at surgery was calculated in 191/236 (81%) RCB2/3 patients (Appendix 8). The TILs increase was ≥20% observed in 38/69 (20%) trastuzumab, 16/43 (23%) lapatinib, 12/33 (36%) combination, and in 1/46 (2%) control patients (*P* = 0.002). Ki67 response was observed in 21/35 (60%) patients with ≥20% TILs increase and in 56/152 (37%) patients without (*P* = 0.012). Having high TILs at surgery seemed to explain improved PFS (*P* = 0.02, Fig. 3D) rather than having a ≥20% TILs increase between baseline and surgery (*P* = 0.16).

### Safety

Sixteen serious adverse events were reported in 14/257 (5%) patients. Six were unrelated to treatment [four allocated trastuzumab alone (part 1 and part 2), and two allocated combination]. Ten were classed as expected serious adverse reactions, occurring in two patients allocated to trastuzumab (part 1 and part 2), five allocated to lapatinib (part 1), and three allocated to combination treatment.

An additional cardiac assessment after treatment but before adjuvant chemotherapy was introduced as of April 2014, affecting 90/127 part 2 patients. The assessment was done on 70/90 part 2 patients and one trastuzumab patient showed an abnormal LVEF of 35%, leading to treatment delay. Further details on safety can be found in Appendix 9.

## Discussion

The EPHOS-B Trial met one of its primary objectives, that 11 days of anti-HER2 therapy, between diagnosis and surgery, without chemotherapy, reduced proliferation, which was seen in all active treatment groups but particularly with the dual-agent combination where a Ki67 decrease greater than 30% was seen in 74% of cancers. Furthermore, some tumors became too small to be analyzed at the time of surgery, and exploratory analysis revealed dual blockade with lapatinib and trastuzumab resulted in 4/65 (6%) of cases having no residual invasive disease in the breast or nodes (pCR) and a further 13/65 (20%) cases with only minimal residual disease (RCB1).

The EPHOS-B trial did not meet its second primary objective of showing an increase in apoptosis in treatment groups, in contrast to a small clinical study that reported increases in apoptosis after 7 days after beginning treatment (16), so potentially, because the on-treatment assessment was performed after 11 days of treatment, in some patients any increase in apoptosis may have been missed. Furthermore, high proliferation values at baseline correlated with higher apoptosis, and, in treatment groups, the fall in proliferation led to a fall in apoptosis, as observed elsewhere (6, 18), so few treated patients had a 30% increase in apoptosis. Although the baseline core biopsies had high numbers of Ki67-positive cells to count, low values of apoptosis rose in control patients due to the greater accuracy of assessment on the surgical excision specimens, but fell with the antiproliferative effect of treatments (19).

Both trastuzumab and lapatinib have been previously shown to inhibit HER2-positive breast cancer proliferation when given before surgery (1, 2, 4). Changes in proliferation biomarkers, including Ki67, predict clinical response and long-term outcome after 2 weeks of endocrine therapy in ER-positive breast cancer (5–7). In our study, several safeguards were in place to enable exploratory associations of biomarkers with long-term outcomes: adjuvant therapy was to be given as per local protocols; Ki67 results were not fed back to investigators; and centralized review of RCB status was done retrospectively. Although adjuvant treatment may be a confounding factor for long-term outcomes, we did not observe differences in adjuvant treatment received between ki67 change groups (see Appendix 2), nor that RCB0 or RCB1 responders received any more or less treatment (see Appendix 3). In these exploratory analyses, patients with Ki67 reductions ≥50% at 11 days had 5-year RFS 99%.

Reporting eradication of primary tumors after only 11 days' dual anti-HER2 blockade therapy is unprecedented. In the NEO-SPHERE trial (9), 18/107 (17%) patients achieved pCR after 4 months' treatment with pertuzumab and trastuzumab and no chemotherapy; for ER and/or PgR-positive tumors, only 3/51 (6%) achieved pCR. In the TBCRC006 study (1), patients received lapatinib and trastuzumab, with ER-positive patients (62%) also receiving letrozole. After 12 weeks' therapy, 17/64 (27%) patients achieved pCR: 8/39 (21%) among ER-positive, 9/25 (36%) among ER-negative. In the WSG-ADAPT study (20), 12-week treatment of HER2-positive/ER-negative cancers with trastuzumab and pertuzumab (without chemotherapy) led to 74% exhibiting Ki67 reductions ≥30%, and 36% pCR. Ki67 nonresponders had an 8% pCR rate. It is worth noting that for neo-adjuvant HER2 trials, tumors were typically over 2 cm in size at trial entry, whereas this was not a requirement in EPHOS-B, and the chance of achieving pCR in the combination group was lower for larger tumors.

In EPHOS-B, 26% combination patients whose cancers regressed (pCR or RCB1) was consistent with 30% pCR seen in the PAMELA study (21) and 27% pCR in TBCRC006 (1) after 12 weeks of neoadjuvant dual agent therapy. EPHOS-B used a trastuzumab-accelerated loading dose (6 mg/kg; ref. 22) combined with lapatinib 1,000 mg to ensure maximal HER2 blockade by 11 days, which may partly account for the earlier responses, as previous neoadjuvant studies used a lower initial doses of trastuzumab (9, 21, 23).

Imaging substudies assessing (18)F-FDG PET/CT at 15 days in the Neo-ALTTO and TBCRC026 trials showed greater SUV_max_ reductions predicted pCR in response to dual anti-HER2 therapy (24, 25). However PET/CT studies are not widely available for clinical practice, whereas Ki67 and tumor response at 11 days are practical for wider implementation in the neoadjuvant setting to predict response on dual anti-HER2 therapy potentially reducing toxicity.

ER and HER2-positive tumors are less likely than ER-negative tumors to have pCR in response to several months of anti-HER2 therapy (9, 23, 26–28). Observing 6% pCR and 20% RCB1 in a population with two-thirds having ER-positive tumors, after 11 days' therapy, was a surprising finding, as there was no evidence that the ER status of the cancer influenced pCR. pCR incidence in ER-positive/HER2-positive cancers is usually lower than in ER-negative/HER2-positive cancers (29). The observed effects on both Ki67 and tumor response in the ER- and HER2-positive cancers may relate to the second anti-HER2 therapy used. Tyrosine kinase inhibitors such as lapatinib interfere with the intercellular tyrosine kinase signaling, which is known to interact with ER signaling. Recent pCR reports in 27% to 44% HER2-positive cancers after 12 to 16 weeks of combination therapy (30, 31) imply that there is a group of patients with HER2 whose primary cancers are highly dependent on HER2 signaling, and their early identification would (if validated in further studies) allow testing of the omission of chemotherapy without detrimental effects on oncologic outcomes.

The WSG-ADAPT (20) trial tested a de-escalation approach following identification of early responders: in largely stage 1 HER2-positive HR-negative breast cancers, after Ki67 assessment at 3 weeks of pertuzumab and trastuzumab, patients were randomized to continued dual agent therapy up to 12 weeks, or combined it with weekly paclitaxel. Nonresponse at 3 weeks predicted lack of pCR at 12 weeks, but addition of paclitaxel in early responders produced 79% pCR, superior to 45% pCR observed when no paclitaxel was added.

The PerELISA (32) neoadjuvant study enrolled mainly patients with stage 2/3 HR-negative HER2-positive cancers that were treated for 2 weeks with an aromatase inhibitor and re-biopsied. Reductions in Ki67 ≥50% allowed treatment with dual-agent pertuzumab and trastuzumab, whereas nonresponders additionally received paclitaxel. After 13 weeks of treatment, pCR and RCB1 occurred in 52% of early responders. If dual antibody therapy can eradicate some HER2-positive breast cancers in less than 2 weeks, a similar approach using a letrozole and dual anti-HER2 therapy from initial biopsy may improve selection of patients who can avoid chemotherapy. These studies imply that a Ki67 reduction >50% to anti-HER2 therapy after 2 weeks of treatment predicts outcome and that approaches to de-escalation will likely differ according to ER status. Our data, taken with these studies, suggest there may be HER2-positive breast cancers that can be eradicated without chemotherapy. Our data add evidence that reductions in Ki67 or pCR/RCB1 (by image-guided biopsy or surgery) after 11 days of treatment potentially allows clinicians to select patients who could receive less chemotherapy, a strategy that needs validation in further studies.

TILs affect RFS and response to therapy, but the effect seems driven by trastuzumab (alone or in an combination): among RCB2/3 trastuzumab patients, a higher Ki67 response was observed when a relevant increase in TILs occurred (62%) compared with those without an increase in TILs (43%, Appendix 7). Moreover, the phenotype of TILs may also alter from suppressor to effector TILs, but we could not assess that on the samples available. The alterations in TILs >20% with the large reduction in tumor proliferation may have produced the tumor shrinkage seen by 11 days.

Early data with trastuzumab, when given concurrently with or after adjuvant chemotherapy, led to concerns about cardiotoxicity, (33) but there were no effects on LVEF in the combination group, and no operations were rescheduled because of cardiac issues in our study. All three neoadjuvant trials using dual-agent HER2 blockade with chemotherapy have not found increased short-term cardiotoxicity. (2, 9, 23) A significant part of the cardiac toxicity reported with anti-HER2 therapy may relate to its use with anthracycline chemotherapy.

The next generation of studies of anti-HER2 therapy in early breast cancer need to address both the potential to reduce chemotherapy in some patients and additional approaches in others, as defined by their demonstrated sensitivity to short-duration anti-HER2 therapies. The data we report on the early disappearance of tumors 11 days after treatment commencement may identify a patient group highly sensitive to the HER2-pathway who can potentially avoid chemotherapy altogether.

## Supplementary Material

Supplementary Data

Supplementary Figure

Supplementary Figure

Supplementary Figure

Supplementary Figure

Supplementary Figure

Supplementary Figure

Supplementary Figure

Supplementary Figure

Supplementary Figure

Supplementary Figure
