# Supplementary material for: Combined Perioperative Lapatinib and Trastuzumab in Early HER2-Positive Breast Cancer Identifies Early Responders: Randomized UK EPHOS-B Trial Long-Term Results
Source: Clin Cancer Res. 2022 Feb 14;28(7):1323–34. doi: 10.1158/1078-0432.CCR-21-3177 (PMC9610457; doi:10.1158/1078-0432.CCR-21-3177)
Supplement: Supplementary Data [file ccr-21-3177_supplementary_data_supp0.pdf]

Appendix - Supplementary material for EPHOS-B manuscript

Table of contents

1 Additional details on trial methods ... 2
2 Adjuvant treatment ... 6
Table 1. Adjuvant treatment, by randomised group (patients eligible to start treatment) ... 6
Table 2. Adjuvant treatment, by ki67 % reduction (patients with paired ki67 or breast pCR) ... 7
3 Disease response – exploratory endpoint ... 8
Table 3. Details of the tumours with RCB0 (pCR) or RCB1 ... 8
Table 4. Logistic regression of disease response (pCR or RCB1) within patients allocated to Combination ... 9
4 Appendix 4 – Sensitivity analyses on ki67 perioperative changes ... 10
Table 5. Sensitivity Ki67 analysis with data imputed for breast pCR patients - Part 1 ... 11
Table 6. Sensitivity Ki67 analysis with data imputed for breast pCR patients - Part 2 ... 11
Table 7. Sensitivity analysis –Unadjusted and adjusted estimates of treatment effects for Ki67 response (>30%) ... 12
Table 8. Exploratory analyses – Prognostic factors for Ki67 falls of 30% or larger ... 13
Table 9. Exploratory analyses - Logistic regression of factors related to Ki67 falls of 50% or larger ... 14
Table 10. ... 14
5 Association between HER2 by FISH (centrally assessed) and trial outcomes ... 15
Table 11. Association of HER2 by FISH and treatment outcomes ... 17
6 Association between Ki67 and apoptosis ... 18
7 Analysis of time to event endpoints ... 19
Table 12. Relapse-free survival events and deaths, by randomised treatment and overall ... 19
Table 13. Relapse Free Survival – randomised treatment effects ... 20
Table 14. Relapse Free Survival – prognostic factors ... 21
8 Appendix 9 – Exploratory analysis of Tumour Lymphocytes Infiltrates (TILs) ... 22
Table 15. Change in TILs from baseline to surgery in patients with RCB2/3 tumours (n=191) ... 23
9 Appendix 10 – Further safety details ... 24
Table 16. Serious Adverse Events observed during peri-operative treatment ... 24

1 Additional details on trial methods

Trial governance:

The trial was approved by the South Birmingham Research Ethics Committee (09/H1208/52) and local research and development offices. The study was co-sponsored by University of Manchester/Manchester University NHS Foundation Trust and The Institute of Cancer Research, and was conducted in accordance with the principles of Good Clinical Practice. All patients provided written informed consent. The Institute of Cancer Research Clinical Trials and Statistics Unit (ICR-CTSU) was responsible for trial management, central statistical monitoring and all analyses. Safety and efficacy data were reviewed regularly by an independent data monitoring committee (IDMC). An independent trial steering committee provided trial oversight on behalf of the funders and sponsors. Both committees approved the trial design adaption between Part-1 and Part-2.

Inclusion criteria

- Women aged ≥18 years.
- HER2 positive (3+ on IHC or amplification proven by FISH*) operable invasive breast cancer diagnosed by core biopsy.
- Planned surgery.
- Liver function tests (LFTs) should be normal for the institution (gamma GT, ALT and alkaline phosphatase). Serum creatinine and bilirubin <2 times the upper limits of normal for the institution, or creatinine clearance >60mL/min. (Marginally abnormal test results should be repeated).
- ECOG performance status 0, 1, or 2 (Karnofsky ≥ 60%).
- Non pregnant and non-lactating with no intention of pregnancy during study treatment. Women of childbearing potential must agree to use adequate non-hormonal contraception for the duration of the treatment phase of the study (adequate contraceptive measures include intra-uterine device, barrier method e.g. diaphragm and condoms used in conjunction with spermicidal jelly). Women of childbearing potential must have a negative blood serum pregnancy test within 28 days prior to randomisation.
- Patients must be candidates for and willing to undergo adjuvant chemotherapy and trastuzumab post-surgery.
- Written informed consent obtained for trial and to donation of tissue and blood samples.

Exclusion criteria

- HER2 negative cancers and those with unknown HER2 status.
- Inoperable breast cancer (T4 category) or suspicion of distant metastases.
- Diagnosis of inflammatory breast cancer.
- Clinical evidence of metastatic disease.
- Prior trastuzumab therapy within the last 12 months or local (radiotherapy) cancer treatments.
- Previous cancer at any other site that has been treated within the last 6 months (except previous basal cell carcinoma and cervical carcinoma in situ)
- Have current active hepatic or biliary disease (with exception of patients with Gilbert's syndrome, asymptomatic gallstones or stable chronic liver disease per investigator assessment).
- Impaired gastro-intestinal function thought sufficient to reduce lapatinib absorption.
- Contra-indicated to receive adjuvant chemotherapy and /or trastuzumab (ECOG performance status >2).
- Known immediate or delayed hypersensitivity, reaction to drugs chemically related to trastuzumab or lapatinib.
- Other concomitant investigational agents or concurrent anti-cancer therapy.
- Use of herbal (alternative) therapies within 2* weeks of study entry (see Appendix 1). NB: vitamin and / or mineral supplements are allowed.

- If patients are taking any of the prohibited medication as listed in Appendix 1.
- Regular use of systemic steroids or other agents that could influence study endpoints (inhaled steroids are allowed).
- Any altered mental state that would preclude obtaining written informed consent.
- Clinically significant cardiac abnormalities or uncontrolled hypertension.
- Previous myocardial infarction, heart failure, or significant angina. Cardiac function should be assessed by physical examination, ECG, and baseline LVEF should be ≥55% as measured by echocardiography or MUGA.

Procedures

Patients receiving peri-operative treatment were to commence adjuvant chemotherapy ≥35 days after the first pre-operative dose of trial treatment, allowing at least 7 days of lapatinib washout. Adjuvant treatment was as per local policy and not to be influenced by EPHOS-B allocated treatment. All patients were to receive further adjuvant trastuzumab treatment after adjuvant chemotherapy. Centres were able to choose at their discretion whether to take into account that some patients had already received up to 4 weeks of peri-operative anti-HER2 therapy when prescribing the duration of their standard adjuvant trastuzumab. Patients with hormone receptor (HR) positive cancers were to be treated with either tamoxifen or an aromatase inhibitor for a minimum of 5 years as per local protocols.

Patients were scheduled to be followed up every 6 months for 2 years after randomization then annually to 10 years, with data collected on cardiac toxicity, clinical examination and any local or metastatic relapse. Following a recommendation from the IDMC to better assess cardiac sequelae caused by the administration of combination anti-HER2 therapies in the peri-operative period, from April 2014, Part-2 patients had an additional ECG and ECHO/MUGA before the start of adjuvant chemotherapy (protocol version 6, Dec 18, 2013).

Assessment of biomarkers

Formalin-fixed, paraffin embedded (FFPE) tumour blocks from diagnostic core biopsy and surgical specimens after pre-operative study therapy were obtained. Hormone receptor status (ER and, when available, progesterone receptor [PgR]) was locally evaluated by immunohistochemistry (IHC): Allred (or Quickscore), percentage of tumour cells or H-score were recorded if available. The cut-off for positivity was ≥1% tumour cells, or Allred/Quickscore ≥3. In Part 1, PR status was also centrally assessed.

HER2 was evaluated locally and judged positive by immunohistochemistry 3+ score or fluorescence in situ hybridization (FISH) amplification. FISH assessment was repeated centrally retrospectively. For both local and central, FISH ascertainment should be done as follows: using a two probe system in which at least 100 cells were counted, the ratio of the HER2 signal to the control centromeric probe on chromosome 17 was derived. This was then categorized into FISH:

- Negative: a HER2/centromeric control probe ratio of less than 1.8
- Borderline negative: between 1.8 and 2
- Borderline positive: between 2 and 2.2
- Positive: 2.2 or greater and grossly amplified >5 where the HER2 signal is clumped and cannot be properly counted but is clearly present in large amounts.

For borderline counts further counting should be performed on more fields but if the ratio remains the same all counts of 2 and above are regarded for treatment purposes as positive and those below as negative.

Baseline and surgery samples were centrally assessed for quality and tumour content, and analysed for Ki67 and apoptosis by IHC. Methods for Ki67 and caspase 3 analyses have been previously described [Leary A et al 2015 Clin Cancer Res 21:2932-40; Dowsett M et al 2001 Cancer Epidemiol Biomarkers Prev 10:961-6; Hadjiloucas I et al 2001 Br J Cancer 85:1522-6] The following antibodies were used: monoclonal antibody HER2, clone 4B5 (Ventana Roche),

Ki67/clone Ki67 MIB-1 (Dako, Glostrup, Denmark, http://www.dako.com), antibody for anti-activated caspase 3 (Cell signaling, New England (UK) Biolabs Ltd, UK).

Scoring for Ki67 and apoptosis on each sample was carried out by at least two observers who were blinded to randomized treatment allocation. The scores were compared and any differences >10% were resolved by double-headed microscopic examination and a consensus was reached. The Ki67 and apoptosis percentage was calculated for each patient as the sum of positive cells counted by all observers divided by the sum of total invasive tumour cells counted by all observers.

Sample size

Original design:

The original design was designed to detect differences in the proportion of patients showing changes in apoptosis (activated caspase 3) and proliferation (Ki67) between control vs lapatinib vs trastuzumab. It was powered based on a rise of 30% or more in apoptosis and/or a fall in proliferation of 30% or more [Mohsin SK et al 2005 J Clin Oncol 23:2460-8]. A total of 6 comparisons were planned; three comparisons between groups: trastuzumab vs control, lapatinib vs control and trastuzumab vs lapatinib, undertaken for the two endpoints: apoptosis and proliferation. Each comparison would have a power of 85% or more with a one-sided p-value of 0.0085 to allow for multiple comparisons, given the following assumptions: only 5% or less of control patients will show a 30% rise, but 30% or more of trastuzumab or lapatinib patients will show such a rise. Such differences could be detected with 85% power. It would be possible to detect differences of 25%, e.g. 55% vs 30% with 85% power when comparing the trastuzumab and lapatinib groups.

Adaptation to current design:

As external evidence became available to support an effect of lapatinib on proliferation, and a general biological effect of both trastuzumab alone and the combination, EPHOS-B Part 2 was designed as a three group randomized trial comparing trastuzumab alone vs. the combination of lapatinib+trastuzumab vs. control. The original sample size of 250 patients was maintained, but the randomization allocation was adjusted a so that a higher proportion of patients were treated with the dual therapy group (2:1:1 ratio). Between-group comparisons are restricted to concurrently randomized patients with no indirect comparisons made between, for example, lapatinib Part-1 with combination Part-2 patients. External evidence allowed us to increase the false positive rates for each of the treatment comparisons (see table below) from 0.85% to 2.5% on a heuristic basis. We therefore accounted only for multiplicity due to having co-primary endpoints (ki67 and apoptosis), but not due to the multiple treatment groups. Indeed, although for each part of the trial, these comparisons share a control, as the hypotheses inform different claims of effectiveness (e.g. T is better than C, L is better than C), adjustment of the type-I error is considered to be unnecessary or too conservative [Freidlin et al 2008 Clinical Cancer Research 14:4368-71; Proschan MA 2000 Controlled Clinical Trials 21: 527-39]. Assuming that a) a 30% increase in apoptosis or a 30% decrease in proliferation may be seen by chance in 5% of control patients, b) clinically important differences are between 30% to 60% of patients showing such responses and c) respecting the old/ new randomization, then with power >80% and one-sided a= 0.025, the following table describes some illustrative amended powering considerations at the time of change in design:

Comparison:	T+L vs. C (part2)	T vs. C (part1&2)	T+L vs. T (part2)	T vs. L (part1)	L vs. C (part1)
a (1-sided)	0.025	0.025	0.025	0.025	0.025
power	0.85	0.90	0.85	0.80	0.85
1st proportion	0.05	0.05	0.3	0.38	0.05
2nd proportion	0.3	0.3	0.6	0.6	0.38
Allocation ratio	2	2	2	1	2
N1=	32	36	36	42	21
N2=	64	72	72	42	42

Statistical analyses:

Percentage change in Ki67 and apoptosis were calculated as [((surgery score+0.1) - (pre-treatment score+0.1))/(pre-treatment score+0.1)]*100. Log fold change was calculated as ln((surgery score+0.1)/(pre-treatment score+0.1)). The constant of 0.1 was added to accommodate cases with a value of 0%. Percentage changes were displayed using waterfall plots and compared between randomized treatment groups using the Mann–Whitney non-parametric test. The proportion of patients responding with a decrease in Ki67/increase in apoptosis of >30%, together with associated 95% confidence intervals, are presented by randomized treatment group. Proportions were compared between randomized treatment groups using Fisher's exact test. Logistic regression was used to estimate unadjusted and adjusted treatment effects for the randomised comparisons in each Part. The same models were used to investigate factors affecting Ki67 response with the pooled dataset. An alternative threshold of response of 50% fall was also explored as pre-specified in the Statistical Analysis Plan (following the response rate used in the MAPLE trial (Leary et al, Clin Cancer Res, 2015 Jul 1;21(13):2932-40. doi: 10.1158/1078-0432.CCR-14-1428). The proportion of patients with pCR in both breast and nodes (RCB0) or with minimal residual disease (RCB1) was calculated within each randomized treatment group.

All randomized patients were included in the analysis of time-to-event endpoints, which was summarized graphically by Kaplan-Meier estimates and groups compared with log-rank tests. Adjusted and unadjusted treatment effects for each part were estimated by Cox proportional hazards models. Association of peri-operative changes with RFS was similarly analysed; Part-1 and Part-2 were combined for this analysis and log-rank tests stratified by treatment group. Cox regression was used to investigate factors affecting RFS in univariate and multivariable models. The proportional hazards assumption was assessed using Schoenfeld residuals and found to hold.

2 Adjuvant treatment

Table 1. Adjuvant treatment, by randomised group (patients eligible to start treatment)

	PART 1						PART 2						Total N=255	
	Trastuz N=56		Lapatinib N=51		Control N=22		Trastuz N=32		Combination N=65		Control N=29			
	No.	%	No.	%	No.	%	No.	%	No.	%	No.	%	No.	%
**Chemotherapy**														
Yes	56	100.00	49	96.1	22	100.0	32	100.0	60	92.3	27	93.1	246	96.50
Docetaxel	*0*	*0*	*1*	*2*	*0*	*0*	*0*	*0*	*1*	*1.5*	*0*	*0*	*2*	*0.8*
Docetaxel+Carboplatin	*10*	*17.9*	*2*	*3.9*	*1*	*4.5*	*5*	*15.6*	*6*	*9.2*	*2*	*6.9*	*26*	*10.2*
Docetaxel+Cyclophosphamide	*1*	*1.8*	*0*	*0*	*0*	*0*	*0*	*0*	*1*	*1.5*	*1*	*3.4*	*3*	*1.2*
Docetaxel+Doxorubicin+Cyclophosphamide	*1*	*1.8*	*1*	*2*	*0*	*0*	*0*	*0*	*0*	*0*	*0*	*0*	*2*	*0.8*
Doxorubicin+Cyclophosphamide	*0*	*0*	*1*	*2*	*0*	*0*	*0*	*0*	*0*	*0*	*0*	*0*	*1*	*0.4*
Epirubicin	*3*	*5.4*	*4*	*7.8*	*4*	*18.2*	*0*	*0*	*0*	*0*	*0*	*0*	*11*	*4.3*
Epirubicin+Cyclophosphamide	*12*	*21.4*	*10*	*19.6*	*4*	*18.2*	*9*	*28.1*	*15*	*23.1*	*10*	*34.5*	*60*	*23.5*
Fluorouracil+Epirubicin+Cyclophosphamide	*25*	*44.6*	*30*	*58.8*	*13*	*59.1*	*16*	*50*	*31*	*47.7*	*13*	*44.8*	*128*	*50.2*
Paclitaxel	*4*	*7.1*	*0*	*0*	*0*	*0*	*2*	*6.3*	*4*	*6.2*	*1*	*3.4*	*11*	*4.3*
Paclitaxel+Carboplatin	*0*	*0*	*0*	*0*	*0*	*0*	*0*	*0*	*2*	*3.1*	*0*	*0*	*2*	*0.8*
Number of cycles - N, Median [IQR]	55	4 [3-6]	49	3 [3-6]	22	3 [3-4]	30	3 [3-6]	59	3 [3-6]	26	3 [3-6]	241	3 [3-6]
No	0	0.00	2	3.9	0	0.0	0	0.0	5	7.7	2	6.9	9	3.5
Patient declined	-	-	*1*	*50.0*	-	-	-	-	*2*	*3.1*	*1*	*3.4*	*4*	*1.6*
Clinician decision	-	-	*1*	*50.0*	-	-	-	-	*2*	*3.1*	-	-	*3*	*0.8*
Received neo-adjuvant chemotherapy	-	-	-	-	-	-	-	-	--	--	*1*	*3.4*	*1*	*0.4*
Reason not given	-	-	-	-	-	-	-	-	*1*	*1.5*	-	-	*1*	*0.4*
p-value (exact test CT (yes/no) vs randomised group)			0.468						0.268					
**Anti-HER2 therapy**														
Trastuzumab	*55*	*98.20*	*48*	*94.1*	*22*	*100.0*	*31*	*96.9*	*59*	*90.8*	*28*	*96.6*	*243*	*95.30*
Lapatinib	*0*	*0.00*	*1*	*2.0*	*0*	*0.0*	*0*	*0.0*	*0*	*0.0*	*0*	*0.0*	*1*	*0.40*
None planned	*0*	*0.00*	*2*	*3.9*	*0*	*0.0*	*1*	*3.1*	*4*	*6.2*	*1*	*3.4*	*8*	*3.10*
Other	*1**	*1.80*	*0*	*0.0*	*0*	*0.0*	*0*	*0.0*	*2***	*3.1*	*0*	*0.0*	*3*	*1.20*
p-value (exact test Anti-HER2 (yes/no) vs randomised group)			0.468						>0.999					
**Endocrine therapy**														
Yes	36	64.3	29	56.90	14	63.6	18	56.3	49	75.4	16	55.2	162	63.5
Tamoxifen	*20*	*35.7*	*16*	*31.40*	*6*	*27.3*	*9*	*28.1*	*20*	*30.80*	*4*	*13.8*	*75*	*29.4*
AI/Tam switch	*6*	*10.7*	*4*	*7.80*	*3*	*13.6*	*4*	*12.5*	*10*	*15.40*	*4*	*13.8*	*31*	*12.2*
AI only	*10*	*17.9*	*9*	*17.70*	*5*	*22.7*	*5*	*15.6*	*19*	*29.20*	*8*	*27.6*	*56*	*22.0*
p-value (Chi2 test endocrine therapy (yes/no) vs randomised group)			0.71						0.067					
**Other treatments**														
Ovarian ablation	0	0.0	0	0.0	0	0.0	0	0.0	1	1.6	0	0.0	1	0.4
Radiotherapy	39	69.6	37	72.6	16	72.7	26	81.3	52	80.0	23	79.3	193	75.7
p-value (Chi2 test radiotherapy (yes/no) vs randomised group)			0.934						0.981					

**Palliative trastuzumab - no set duration; ** 2pt: trastuzumab & lapatinib*

Table 2. Adjuvant treatment, by ki67 % reduction (patients with paired ki67 or breast pCR)

Only patients with paired ki67 or with breast PCR response included in the analysis. Patients with breast pCR were considered to have 100% reduction.

	>50% Ki67 fall		10-50% ki67 fall		<10% or no ki67 fall		Total (n=231)	
	**No.**	**%**	**No.**	**%**	**No.**	**%**	**No.**	**%**
**Chemotherapy**								
Yes	69	95.80	75	97.4	80	97.7	224	97.0
Docetaxel	*1*	*1.4*	*1*	*1.3*	*0*	*0*	*2*	*0.9*
Docetaxel+Carboplatin	*8*	*11.1*	*7*	*9.1*	*8*	*9.8*	*23*	*10*
Docetaxel+Cyclophosphamide	*1*	*1.4*	*0*	*0*	*1*	*1.2*	*2*	*0.9*
Docetaxel+Doxorubicin+Cyclophosphamide	*0*	*0*	*2*	*2.6*	*0*	*0*	*2*	*0.9*
Doxorubicin+Cyclophosphamide	*0*	*0*	*1*	*1.3*	*0*	*0*	*1*	*0.4*
Epirubicin	*3*	*4.2*	*4*	*5.2*	*3*	*3.7*	*10*	*4.3*
Epirubicin+Cyclophosphamide	*14*	*19.4*	*19*	*24.7*	*22*	*26.8*	*55*	*23.8*
Fluorouracil+Epirubicin+Cyclophosphamide	*36*	*50*	*37*	*48.1*	*44*	*53.7*	*117*	*50.7*
Paclitaxel	*5*	*6.9*	*4*	*5.2*	*2*	*2.4*	*11*	*4.8*
Paclitaxel+Carboplatin	*1*	*1.4*	*0*	*0*	*0*	*0*	*1*	*0.4*
Number of cycles - N, Median [IQR]	68	3 [3-6]	72	3 [3-6]	79	3 [3-6]	219	3 [3-6]
No	3	4.2	2	2.6	2	2.4	7	3.0
Patient declined	*1*	*1.4*	*0*	*0.0*	*2*	*2.4*	*3*	*1.3*
Clinician decision	*1*	*1.4*	*1*	*1.3*	-	-	*2*	*0.9*
Received neo-adjuvant chemotherapy	-	-	-	-	-	-	-	-
Reason not given	*1*	*1.4*	*1*	*1.3*	-	-	*2*	*0.9*
p-value (exact test use CT (yes/no) vs Ki67 group)	0.6							
**Anti-HER2 therapy**								
Trastuzumab	*68*	*94.4*	*73*	*94.8*	*80*	*97.6*	*221*	*95.7*
Lapatinib	*1*	*1.4*	*0*	*0.0*	*0*	*0.0*	*1*	*0.4*
Other	*1*	*1.4*	*2*	*2.6*	*0*	*0.0*	*3*	*1.3*
None planned	*2*	*2.8*	*2*	*2.6*	*2*	*2.4*	*6*	*2.6*
p-value (exact test use Anti-HER2 (yes/no) vs Ki67 group)	>0.999							
**Endocrine therapy**								
Yes	50	69.4	51	66.2	46	56.1	147	63.6
Tamoxifen	*26*	*36.1*	*28*	*36.4*	*17*	*20.7*	*71*	*30.7*
AI/Tam switch	*10*	*13.9*	*9*	*11.7*	*9*	*11.0*	*28*	*12.1*
AI only	*14*	*19.4*	*14*	*18.2*	*20*	*24.4*	*48*	*20.8*
p-value (Chi2 test use endocrine therapy (yes/no) vs ki67 group)	0.193							
**Other treatments**								
Ovarian ablation	0	0.0	1	1.3	0	0.0	1	0.4
Radiotherapy	60	83.3	61	79.2	56	68.3	177	76.6
p-value (Chi2 test use radiotherapy (yes/no) vs ki67 group)	0.08							

3 Disease response – exploratory endpoint

Table 3. Details of the tumours with RCB0 (pCR) or RCB1

Patient	Treatment group	Tumour size (cm) (clinical/ radiological)	Baseline sample						Surgery	Nodes involved/ examined	RCB	Adjuvant treatment	RFS event	Follow-up time (OS)*
			Grade	HER2	Ampl Ratio (FISH)	ER	PgR	ER/PgR (Allred)						
1	Combination (Part 2)	2	2	3+ (IHC)	6.41	Positive	Negative	8/2	Mx, SNB	0/2	RCB0	CT H T RT	None	96
2	Combination (Part 2)	0.9	2	3+ (IHC)	10.15	Positive	Negative	4/0	WLE, SNB	0/2	RCB0	CT H AI/T	None	72
3	Combination (Part 2)	2.7	3	3+ (IHC)	17.2	Positive	Negative	4/0	WLE, SNB	0/2	RCB0	CT H RT	None	73
4	Combination (Part 2)	1.6	3	3+ (IHC)	2.46	Positive	Negative	7/0	Mx, SNB	0/2	RCB0	CT H AI RT	None	68
5	Trastuzumab (Part 1)	2.8	2	2+ (FISH)	7.12	Positive	Positive	7/8	Mx, ANC	0/8	RCB0	CT H RT	None	73
6	Trastuzumab (Part 2)	2	3	3+ (IHC)	2.08	Negative	Negative	0/0	WLE, SNB	0/5	RCB0	CT H T	None	62
7	Combination (Part 2)	1.4	3	3+ (IHC)	9.35	Positive	Positive	6/8	WLE, SNB	0/3	RCB1	CT H T RT	None	60
8	Combination (Part 2)	1.8	2	3+ (IHC)	8.66	Negative	Negative	0/0	Mx, ANC	0/11	RCB1	CT H	None	72
9	Combination (Part 2)	1.4	2	3+ (IHC)	4.6	Positive	Positive	7/5	WLE, SNB	0/3	RCB1	**AI RT****	None	71
10	Combination (Part 2)	1.7	3	3+ (IHC)	5.76	Negative	Negative	0/0	WLE, SNB	0/5	RCB1	CT H RT	None	63
11	Combination (Part 2)	0.5	3	3+ (IHC)	2.05	Negative	Negative	0/0	Mx, SNB	0/4	RCB1	CT H	Contr. BC	74
12	Combination (Part 2)	1.2	3	3+ (IHC)	7.19	Positive	Negative	4/0	Mx, SNB	0/1	RCB1	CT H AI	None	76
13	Combination (Part 2)	1.3	Unknown	3+ (IHC)	15.9	Positive	Negative	7/0	Mx, SNB	0/1	RCB1	CT H AI	None	64
14	Combination (Part 2)	1.4	1	3+ (IHC)	10.3	Positive	Positive	7/7	WLE, SNB	0/1	RCB1	CT H+L AI RT	None	61
15	Combination (Part 2)	2	3	3+ (IHC)	6.4	Positive	Unknown	7/-	WLE, SNB	0/2	RCB1	CT H AI/T RT	None	68
16	Combination (Part 2)	0.8	2	2+ (FISH)	2.19	Positive	Unknown	7/-	WLE, SNB	0/1	RCB1	CT H AI/T RT	None	66
17	Combination (Part 2)	4.5	2	3+ (IHC)	5.8	Positive	Unknown	8/-	WLE, ANC	1/18	RCB1	CT H AI/T RT	None	54
18	Combination (Part 2)	1	3	3+ (IHC)	9.72	Negative	Negative	0/0	WLE, SNB	0/2	RCB1	CT H+L RT	None	71
19	Combination (Part 2)	0.9	3	3+ (IHC)	6.48	Positive	Positive	6/6	WLE, SNB	0/1	RCB1	CT H T RT	Local***	71

HER2= human epidermal growth factor receptor 2; ER= oestrogen receptor; PgR= progesterone receptor; RCB= Residual Cancer Burden class; IHC= immunohistochemistry; FISH= fluorescence in situ hybridization; Mx= mastectomy; WLE= wide local excision; ANC= axillary node clearance (level 2/3 dissection); SNB= sentinel lymph node biopsy, CT=chemotherapy, H=Herceptin, H+L=Herceptin+Lapatinib, T=Tamoxifen, AI=aromatase inhibitor, AI/T=AI/Tamoxifen switch, RT=radiotherapy, Contr.BC= Contralateral breast cancer.

Allred scores of 3 or above define positive ER/PgR. No other pCR or RCB1 were observed in the lapatinib or control groups. *All patients alive at end of follow-up. **Patient declined CT and Herceptin. **Local recurrence followed by distant recurrence>1.5 years later

Logistic regression was used to investigate factors associated with RCB1/pCR within the combination group. Analysis includes 57/65 combination patients with full data available for all factors considered below (patients with missing PgR are still included in a missing category). Two patients excluded have missing tumour grade as this was not assessed on core biopsy at baseline, and further 7 patients had missing amplification Ratio on FISH. Neither of the patients not included had an RCB1/pCR. An odds ratio of >1 indicates an increased odds of response (either pCR or RCB1) as compared to the reference group, while an odds ratio <1 indicates a decreased odds of response.

Table 4. Logistic regression of disease response (pCR or RCB1) within patients allocated to Combination

		Responses* / No.	Univariate			Multivariable		
			Odds Ratio	95% CI	p-value	Odds Ratio	95% CI	p-value
ER status (local)	Negative	4/14		1 (ref)			1 (ref)	
	Positive	12/43	0.97	0.25, 3.67	0.96	2.51	0.40, 15.90	0.33
PgR status (local)	Negative	9/24		1 (ref)			1 (ref)	
	Positive	4/16	0.56	0.14, 2.26	LR test	0.26	0.04, 1.67	LR test
	Missing	3/17	0.36	0.08. 1.59	0.36	0.15	0.02, 1.07	0.36
Tumour grade	1-2	7/23		1 (ref)			1 (ref)	
	3	9/34	0.82	0.26, 2.65	0.74	1.07	0.23, 5.01	0.94
Tumour size	≤2cm	14/40		1 (ref)			1 (ref)	
	>2cm	2/17	0.25	0.05, 1.24	0.090	0.19	0.03, 1.14	0.070
Age	Continuous	16/57	1.03	0.97, 1.09	0.33	1.06	0.98, 1.13	0.13
Baseline Ki67	Continuous	16/57	0.98	0.94, 1.02	0.23	0.97	0.92, 1.03	0.29
HER2 amplification ratio	Continuous	16/57	0.95	0.84, 1.09	0.48	0.96	0.82, 1.11	0.57

4 Appendix 4 – Sensitivity analyses on ki67 perioperative changes

4.1 Absolute change in Ki67

Figure 1. Line plot of change in ki67 by treatment group (Part 1& Part 2)

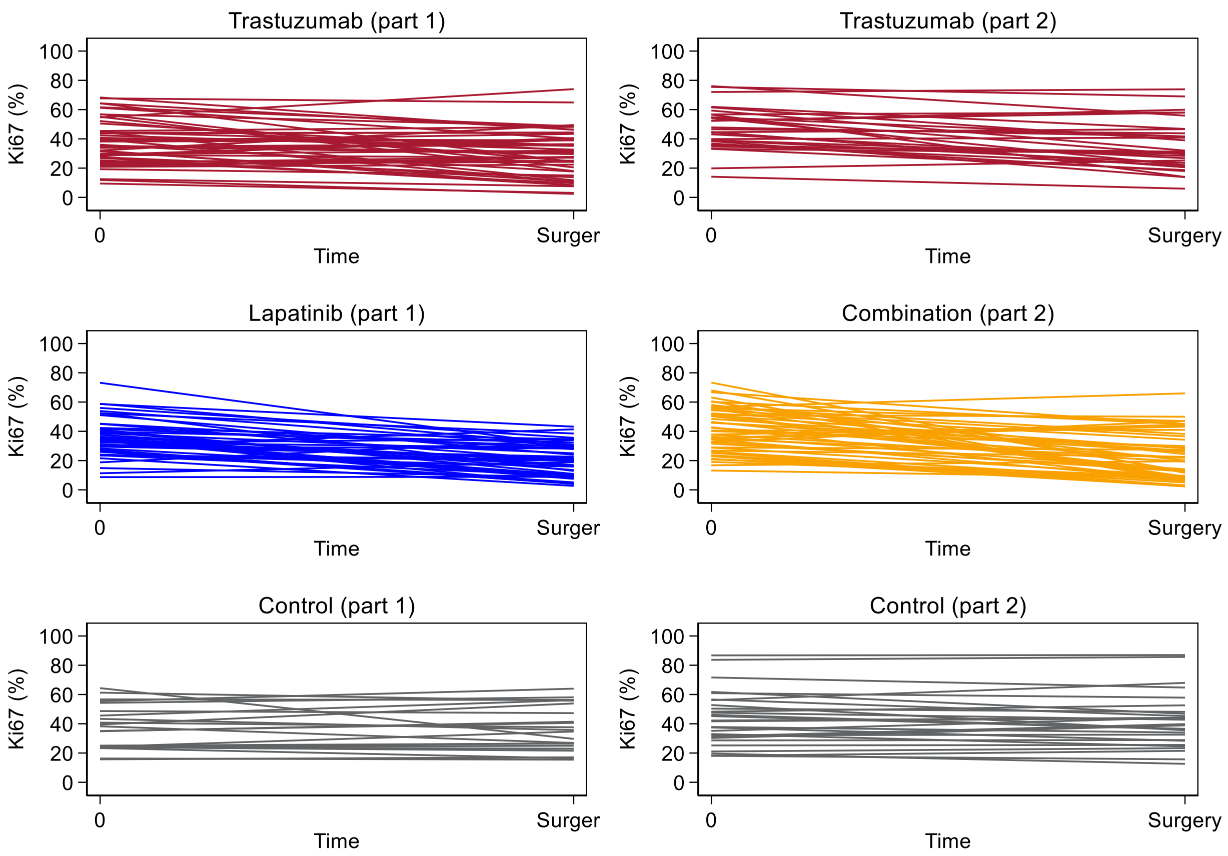

4.2 Sensitivity analysis - Imputing surgery Ki67 for patients with breast pCR

Analyses were repeated including patients who had a breast pCR (cases with pCR or 0% cellularity but nodal involvement) with an imputed surgery Ki67 of 0%, and therefore a percentage change of -100%. This adds an additional 7 evaluable patients to the analysis (1 Part-1 trastuzumab; 6 Part-2 combination).

Table 5. Sensitivity Ki67 analysis with data imputed for breast pCR patients - Part 1

	**PART 1**		
	**Trastuzumab**	**Lapatinib**	**Control**
N with paired Ki67 data	50	44	22
Baseline Ki67 (median, IQR)	35.3 (27.0 - 45.4)	34.2 (27.2 - 42.0)	36.7 (24.0 - 48.7)
Surgery Ki67 (median, IQR)	30.2 (17.7 - 40.1)	20.0 (10.0 - 29.7)	35.2 (25.0 - 53.9)
Percentage change (median, IQR)	-14.7 (-52.8 - 5.6)	-43.0 (-67.9 - -21.1)	1.7 (-9.0 - 15.3)
Logfold change (median, IQR)	-0.16 (-0.75 - 0.05)	-0.56 (-1.14 - -0.24)	0.02 (-0.09 - 0.14)
Mann-Whitney test for % change - Lapatinib vs. Control		p < 0.0001	
Mann-Whitney test for % change - Lapatinib vs. Trastuzumab	p = 0.007		
Response (Ki67 decrease >30%) - N(%)	19 (38.0%)	29 (65.9%)	1 (4.5%)
Fishers exact test - Lapatinib vs. Control		p < 0.0001	
Fishers exact test - Lapatinib vs. Trastuzumab	p = 0.008		

Table 6. Sensitivity Ki67 analysis with data imputed for breast pCR patients - Part 2

	**PART 2**		
	**Trastuzumab**	**Combination**	**Control**
N with paired Ki67 data	31	55	28
Baseline Ki67 (median, IQR)	44.6 (36.8 - 56.6)	40.2 (28.9 - 55.3)	42.0 (30.9 - 54.5)
Surgery Ki67 (median, IQR)	30.3 (22.0 - 46.7)	14.0 (6.7 - 29.9)	39.4 (28.6 - 47.5)
Percentage change (median, IQR)	-26.5 (-46.3 - -5.9)	-57.5 (-84.0 - -32.3)	-2.0 (-19.9 - 7.0)
Logfold change (median, IQR)	-0.31 (-0.62 - -0.06)	-0.86 (-1.84 - -0.39)	-0.02 (-0.22 - 0.07)
Mann-Whitney test for % change - Combination vs. Control		p < 0.0001	
Mann-Whitney test for % change- Combination vs. Trastuzumab	p = 0.0008		
Response (Ki67 decrease >30%) - N(%)	14 (45.2%)	42 (76.4%)	2 (7.1%)
Fishers exact test - Combination vs. Control		p < 0.0001	
Fishers exact test - Combination vs. Trastuzumab	p = 0.005		

4.3 Adjusted treatment effects for Ki67 response of >30% (randomised comparison)

Unadjusted and adjusted estimates of treatment effect on the primary endpoint were obtained by logistic regression model. These illustrate that in Part-2 the unadjusted odds ratio (OR) was 32.3 (95% CI 6.7 – 156.3, p<0.001) for a Ki67 response in patients allocated to combination treatment compared with control, indicating a significantly greater effect on Ki67 with combination anti-HER2 treatment vs control. In multivariable analysis, the equivalent odds ratio was 35.9 (95% CI 6.8 - 189.9 p<0.001), indicating this effect remained after adjustment for other factors (ER status, PgR status, tumour grade, size, age and baseline Ki67). A similar large effect of lapatinib vs control is observed in Part-1. ER and PgR status, tumour grade, tumour size and age were not related to Ki67 decrease in Part-1 or Part-2.

Table 7. Sensitivity analysis –Unadjusted and adjusted estimates of treatment effects for Ki67 response (>30%)

		Responses / No.	Unadjusted			Adjusted		
			**Odds Ratio**	**95% CI**	**p-value**	**Odds Ratio**	**95% CI**	**p-value**
**Part 1**								
Treatment	Control	1/22		1 (ref)			1 (ref)	
	Trastuzumab	17/44	13.2	1.63, 107.53	0.02	14.67	1.70, 126.64	0.02
	Lapatinib	27/38	51.5	6.16, 431.61	<0.001	75.35	7.98, 711.68	<0.001
**Part 2**								
Treatment	Control	2/26		1 (ref)			1 (ref)	
	Trastuzumab	13/30	9.18	1.83, 46.05	0.007	10.43	1.86, 58.54	0.008
	Combination	35/48	32.31	6.68, 156.34	<0.001	35.86	6.77, 189.87	<0.001

4.4 Exploratory analysis - factors associated with Ki67 response (defined by >30% or >50%)

Factors associated with Ki67 relative changes have been investigated by means of univariate and multivariable logistic regression models in the full set of patients. We have explored the primary endpoint (Ki67 falls of 30% or larger) and also an alternative cut-off (pre-specified in the SAP) of 50% or larger. Baseline prognostic factors (ER status, PgR status, grade, size and age), FISH HER2 amplification ratio and antiher2 therapy received (T, L, T+L) or not (C) were considered; baseline Ki67 was kept in the multivariable models given the outcome represent relative % change. A full model with all factors considered, and a reduced model with only those factors that present p-values<0.1 in the univariate models, are presented. An odds ratio (OR) of >1 indicates an increased rate of response compared to the reference group, while an OR <1 indicates a decreased response rate observed. For part 1 data, central PgR status was used in cases where local PgR testing was not performed. Central PgR data is not currently available for Part 2, so a missing category is kept in the model.). Otherwise, only patients with complete baseline data have been included in the models.

Table 8. Exploratory analyses – Prognostic factors for Ki67 falls of 30% or larger

		Responses / No.	Univariate			Multivariable - full			Multivariable – reduced		
			**Odds Ratio**	**95% CI**	**p-value**	**Odds Ratio**	**95% CI**	**p-value**	**Odds Ratio**	**95% CI**	**p-value**
**Response: Ki67 relative change >30% (yes vs no)**											
Treatment	No treatment	3/48		1 (ref)			1 (ref)			1 (ref)	
	AntiHER2 treatment	99/164	22.85	6.81, 76.6	<0.001	24.52	7.12, 84.46	<0.001	23.67	6.94, 80.77	<0.001
ER status (local)	Negative	26/66		1 (ref)			1 (ref)			1 (ref)	
	Positive	76/146	1.67	0.93, 3.02	0.089	1.52	0.64, 3.63	0.34	1.52	0.64, 3.59	0.34
PgR status (local)	Negative	41/100		1 (ref)			1 (ref)			1 (ref)	
	Positive	41/81	1.48	0.82, 2.67	LR test	1.11	0.46, 2.65	LR test	1.07	0.46, 2.54	LR test
	Missing	20/31	2.62	1.14, 6.04	0.06	2.23	0.74, 6.65	0.29	2.30	0.77, 6.83	0.25
Tumour grade*	1-2	47/93		1 (ref)			1 (ref)			--	
	3	55/119	0.84	0.49, 1.45	0.53	0.86	0.43, 1.72	0.66	--	--	--
Tumour size	≤2cm	56/123		1 (ref)			1 (ref)			--	
	>2cm	43/89	1.28	0.74, 2.21	0.38	1.07	0.57, 2.01	0.84	--	--	--
Age	Continuous	102/212	0.99	0.97, 1.02	0.72	1.01	0.98, 1.04	0.52	--	--	--
Baseline Ki67	Continuous	102/212	1.00	0.98, 1.01	0.97	1.01	0.99, 1.03	0.41	1.01	0.99, 1.03	0.46
HER2	Continuous	102/212	1.05	0.99, 1.12	0.091	1.06	0.99, 1.14	0.11	1.06	0.99, 1.14	0.11

Table 9. Exploratory analyses - Logistic regression of factors related to Ki67 falls of 50% or larger

Table 10.		Responses / No.	Univariate			Multivariable - full			Multivariable – reduced		
			Odds Ratio	95% CI	p-value	Odds Ratio	95% CI	p-value	Odds Ratio	95% CI	p-value
**Response: Ki67 relative change >50% (yes vs no)**											
Treatment	No treatment	1/48		1 (ref)			1 (ref)			1 (ref)	
	AntiHER2 treatment	67/164	32.46	4.37, 241.08	0.001	36.77	4.87, 277.86	<0.001	34.66	4.64, 259.24	0.001
ER status (local)	Negative	19/66		1 (ref)			1 (ref)			--	
	Positive	49/146	1.25	0.66, 2.36	0.49	0.88	0.35, 2.19	0.78	--	--	--
PgR status (local)	Negative	28/100		1 (ref)			1 (ref)			--	
	Positive	27/81	1.29	0.68, 2.43	LR test	1.07	0.43, 2.65	LR test		--	--
	Missing	13/31	1.86	0.81, 4.29	0.34	1.44	0.51, 4.11	0.76		--	
Tumour grade*	1-2	38/93		1 (ref)			1 (ref)			1 (ref)	
	3	30/119	0.69	0.46, 1.04	0.080	0.45	0.22, 0.91	0.027	0.44	0.22, 0.87	0.018
Tumour size	≤2cm	40/123		1 (ref)			1 (ref)			--	
	>2cm	28/89	0.95	0.53, 1.71	0.87	0.81	0.42, 1.54	0.51	--	--	--
Age	Continuous	68/212	0.99	0.97, 1.02	0.69	1.00	0.97, 1.04	0.88	--	--	--
Baseline Ki67	Continuous	68/212	0.99	0.97, 1.01	0.27	1.00	0.98, 1.02	0.99	1.00	0.98, 1.02	0.95
HER2	Continuous	68/212	1.04	0.98, 1.12	0.16	1.05	0.98, 1.13	0.19	--	--	--

5 Association between HER2 by FISH (centrally assessed) and trial outcomes

HER2 amplification ratio by FISH (centrally assessed) correlated with change in Ki67 in the trastuzumab group (Part-2 p=0.008, Part-1 and 2 combined p=0.04).

Group	n	Rho	p-value
T (P1)	49	-0.1502	0.3029
L (P1)	42	-0.0987	0.5339
C (P1)	22	0.1236	0.5836
**T (P2)**	**31**	**-0.4645**	**0.0085**
T+L (P2)	46	-0.2952	0.0464
C (P2)	28	0.1662	0.3980
**T (P1&2)**	**80**	**-0.2358**	**0.0352**
C (P1&2)	50	0.1172	0.4178

Pearson correlation (Rho) between log-fold change in Ki67 and amplification ratio within trastuzumab (part 2) and combined trastuzumab groups statistically significant (p=0.008 and p=0.04 respectively). No other statistically significant correlations observed

Figure 2. Amplification ratio vs log fold change in Ki67, by randomized treatment group

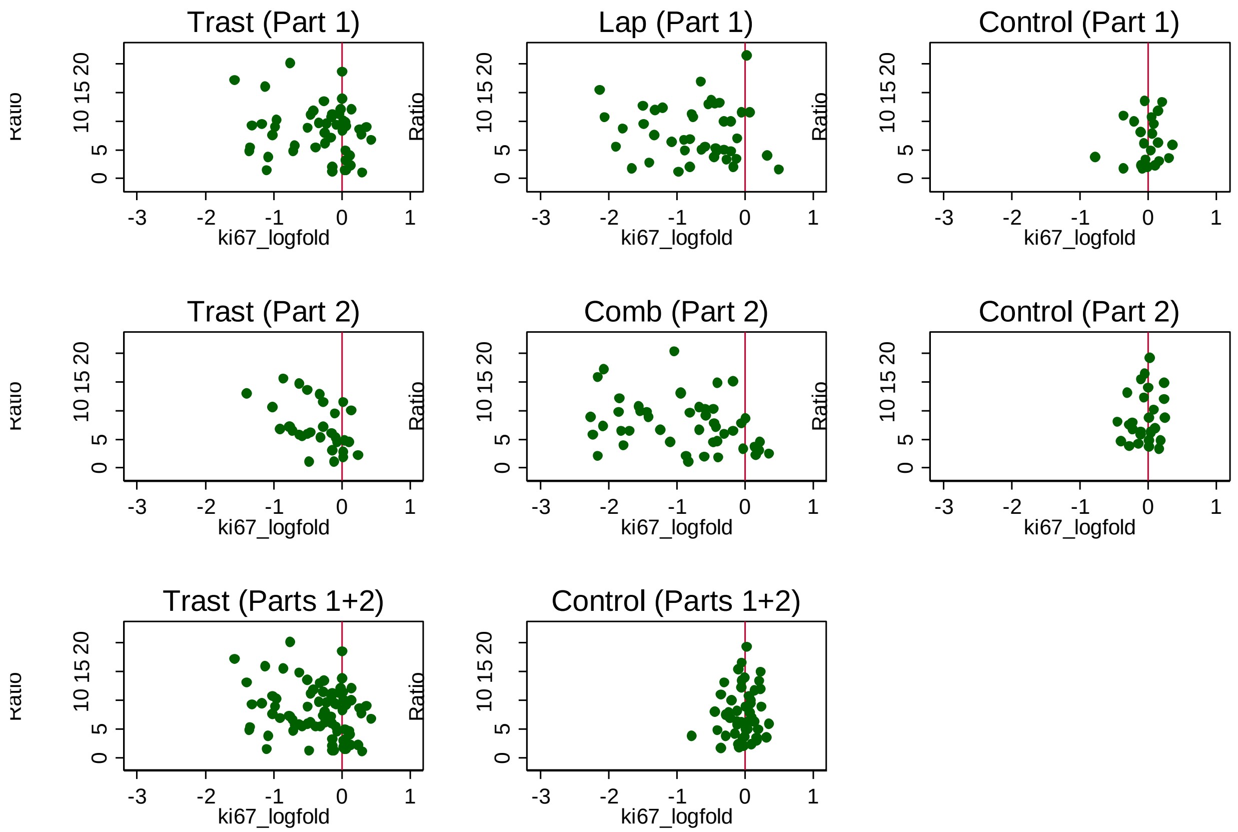

Figure 3. HER2 by FISH and Ki67 response (fall >30%)

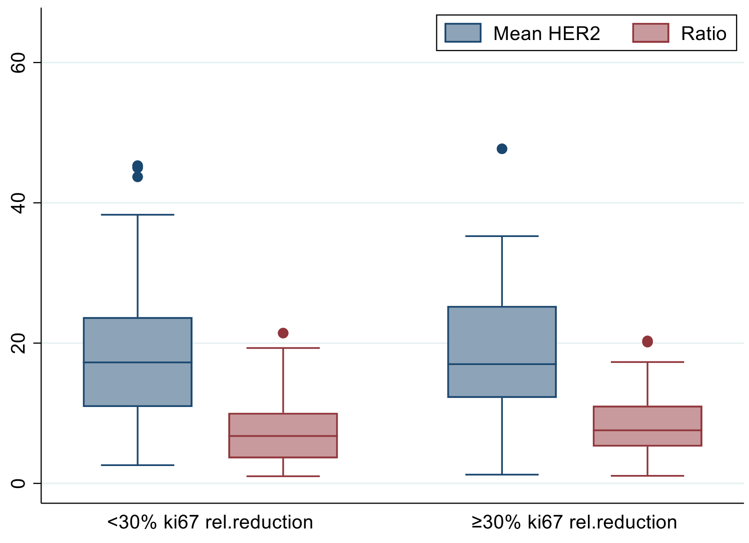

Figure 4. HER2 by FISH and disease response

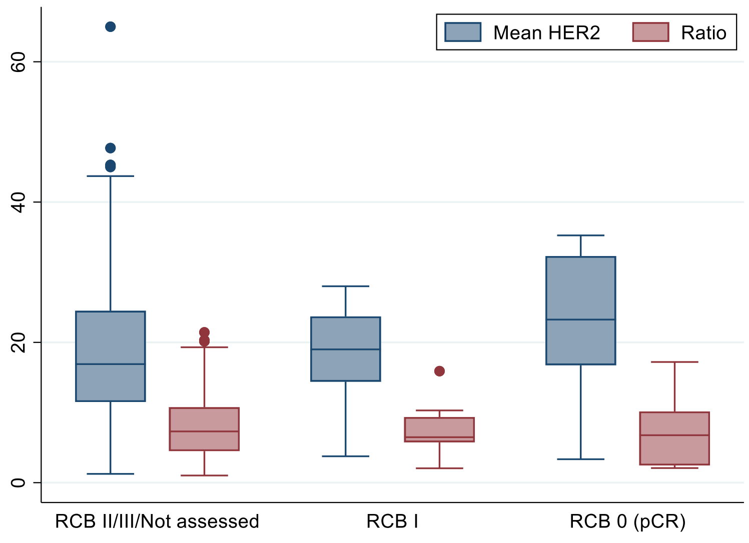

Figure 5. HER2 by FISH and Relapse Free Survival

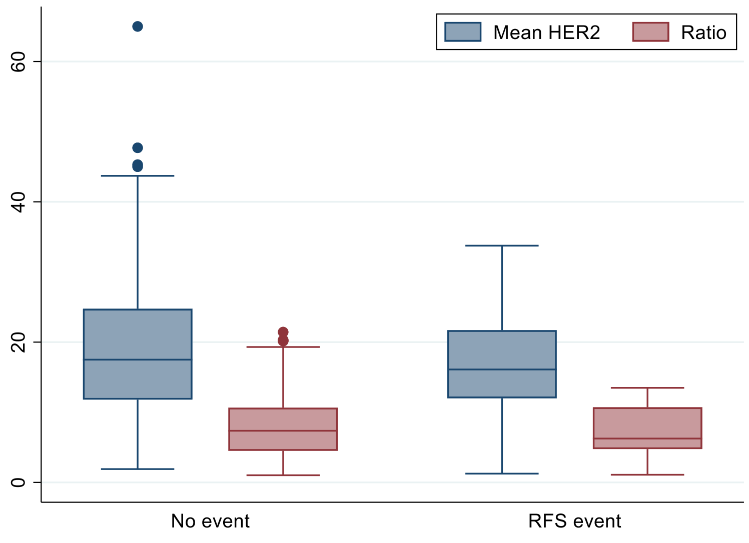

We did not observe any association between HER2 by FISH and trial outcomes. For the amplification ratio, when looking into more detailed I the borderline significance difference between Ki67 responders and not-responders, these were found in the trastuzumab (p=0.053) and combination (p=0.048) Part 2 groups, just as the correlation between HER2 by FISH and log-fold change in Ki67 showed.

Table 11. Association of HER2 by FISH and treatment outcomes

	Mean Her2			Amplification ratio		
	**N**	**Median**	**25-75**	**N**	**Median**	**25-75**
%change Ki67 <30%	118	17.25	10.9-23.7	119	6.76	3.59-10.06
%change Ki67 >=30%	107	17	12.2-25.3	107	7.57	5.25-11.07
p-value		0.75			0.0763	
RCB0	227	16.9	11.5-24.5	228	7.31	4.50-10.75
RCBI	13	19	14.4-23.7	13	6.48	5.76-9.35
RCB2/3	6	23.3	16.75-32.3	6	6.76	2.46-10.15
p-value		0.49			0.56	
No RFS event	221	17.5	11.8-24.75	221	7.36	4.51-10.65
RFS event	25	16.1	12-21.7	26	6.26	4.75-10.7
p-value		0.50			0.50	

p-values – non-parametric tests to compare continuous variables

6 Association between Ki67 and apoptosis

We have explored the association between Ki67 and apoptosis scores at baseline, and for the corresponding %change from baseline within each treatment group.

The baseline biomarker values (across all groups) correlate positively: i.e., greater proportion of Ki67 cells tend to correlate with greater proportion of apoptosis cells at baseline (linear regression, slope p-value<0.001).

Figure 6. Baseline Ki67 by baseline apoptosis (all groups, by part)

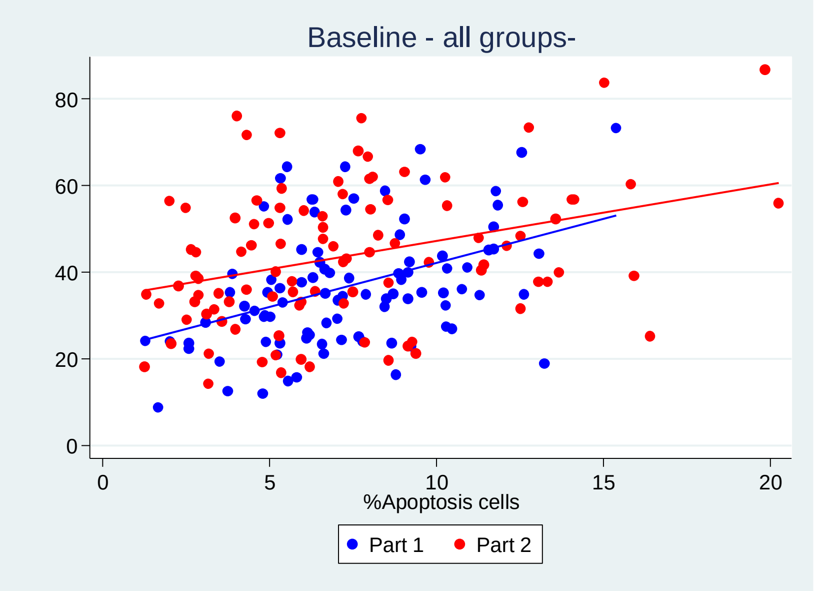

When it comes to association between %change in Ki67 and %change in apoptosis within each treatment group, there is a significant linear association in Part 2 Combination group (p=0.034); no other significant associations present.

Figure 7. % change in Ki67 by %change in apoptosis (all groups)

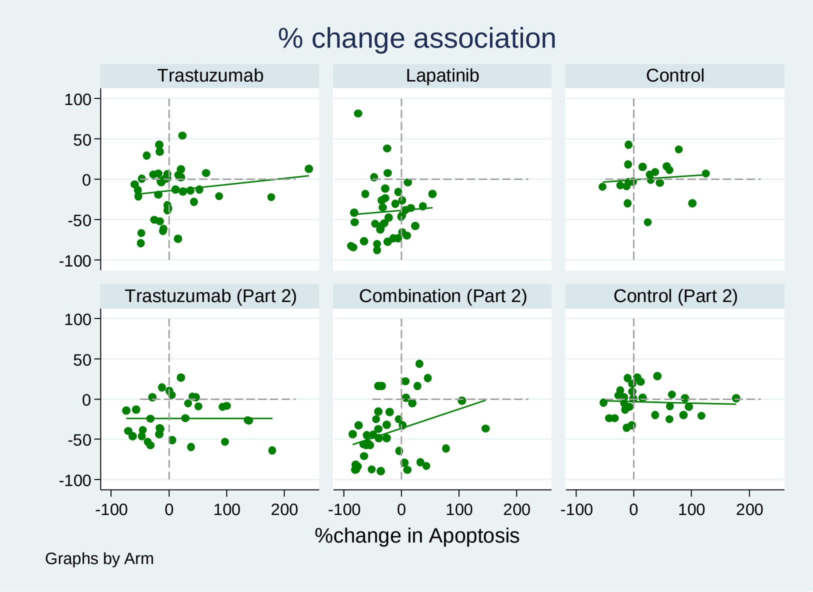

7 Analysis of time to event endpoints

Table 12. Relapse-free survival events and deaths, by randomised treatment and overall

Event type	PART 1						PART 2						Total N=257	
	Trastuz N=57		Lapatinib N=51		Control N=22		Trastuz N=32		Combi nation N=66		Control N=29			
	No.	%	No.	%	No.	%	No.	%	No.	%	No.	%	No.	%
**Relapse Free Survival (RFS)**														
Event contributing to RFS endpoint	7	12.3	5	9.8	1	4.6	7	21.9	5	7.6	3	10.3	28	10.9
Local recurrence (isolated)[(1)]	*0*	-	*0*	-	*0*	-	*3*	*9.4*	*2*	*3.0*	*0*	*0*	*5*	*2.3*
Distant recurrence[(2)]	*7*	*12.3*	*5*	*9.8*	*1*	*4.6*	*4*	*12.5*	*3*	*4.6*	*3*	*10.3*	*23*	*7.0*
Censored observations for RFS	50	87.7	46	90.2	21	95.5	25	78.1	61	92.4	26	89.7	229	89.1
Breast second primary cancer	*0*	-	*1*	*2.0*	*0*	-	*0*	-	*2*	*3.0*	*0*	-	*3*	*1.2*
Non-breast second primary cancer[(3)]	*1*	*1.8*	*2*	*3.9*	*0*	-	*1*	*3.1*	*1*	*1.5*	*1*	*3.5*	*6*	*2.3*
No event observed	*49*	*86*	*43*	*84.3*	*21*	*95.5*	*24*	*75.0*	*58*	*87.9*	*25*	*86.2*	*220*	*85.6*
**Overall Survival (OS)**														
Deaths[(4)]	6	10.5	4	7.8	1	4.5	4	12.5	1	1.5	3	10.3	19	7.4

(1) In 2 cases (1 Part 2 trastuzumab, 1 combination), distant recurrence occurred more than 6weeks later

(2) Include 1 case where distant recurrence was reported within 6 weeks of local recurrence (Part-1 lapatinib) and 1 case were local recurrence was reported >6 weeks later (Part-2 control)

(3) Exclude basal cell carcinomas

(4) 18 deaths due to breast cancer following distant recurrence; 1 intercurrent death (Metastatic gall bladder cancer/liver mets) in absence of breast cancer recurrence (Part-2 control).

7.1 Adjusted treatment effect (randomised comparisons, by part)

Treatment effects for relapse free survival for each part are estimated by means of a Cox proportional hazards model – these are presented unadjusted, and adjusted by known baseline prognostic factors (ER status, PgR status, grade, size and age) and Ki67 response. Analyses are performed separately for part 1 (L vs C, T vs L) and part 2 (T+L vs C, T+L vs T), and then combined (for T vs C). Only patients with complete baseline data have been included in the models.

A hazard ratio (HR) of >1 indicates an increased risk of relapse as compared to the reference group, while a HR <1 indicates a decreased risk of relapse. Patients with pCR are considered Ki67 responders.

Table 13. Relapse Free Survival – randomised treatment effects

		Events / patients	Unadjusted effect			Adjusted effect		
			**HR**	**95% CI**	**p-value**	**HR**	**95% CI**	**p-value**
**Part 1**								
Treatment	Lapatinib	5/38		1 (ref)			1 (ref)	
	Trastuzumab	7/45	1.17	0.37, 3.70	0.78	1.09	0.29, 4.09	0.90
	Control	1/22	0.32	0.04, 2.71	0.29	0.14	0.02, 1.28	0.082
**Part 2**								
Treatment	Combination	4/54		1 (ref)			1 (ref)	
	Trastuzumab	7/31	3.16	0.92, 10.79	0.067	2.29	0.57, 9.13	0.24
	Control	2/26	1.06	0.19, 5.77	0.95	0.93	0.15, 5.62	0.93
**Part 1 + Part 2**								
Treatment	Trastuzumab	5/38		1 (ref)			1 (ref)	
	Control	1/22	3.04	0.87, 10.58	0.08	3.70	0.95, 14.42	0.059

7.2 Factors associated with Relapse Free Survival

Factors associated with relapsed free survival have been investigated by means of univariate and multivariable Cox proportional hazards model in the full set of patients. Baseline prognostic factors (ER status, PgR status, grade, size and age), Ki67 change at surgery, and FISH HER2 amplification ratio have been explored, and antiher2 therapy received (T, L, T+L) or not adjusted for as a stratification factor. Only patients with complete baseline data have been included in the models. **Out of 182 with complete data, only 25 events have been observed – results of the complete multivariable models may be over parametrised and results should be taken with caution.** A full model with all factors considered, and a reduced model with only those factors that present p-values<0.1 in the univariate models are presented. A hazard ratio (HR) of >1 indicates an increased risk of relapse as compared to the reference group, while a HR <1 indicates a decreased risk of relapse. Patients with pCR are considered to have 100% fall in Ki67. For part 1 data, central PgR status was used in cases where local PgR testing was not performed. Central PgR data is not currently available for Part 2.

Table 14. Relapse Free Survival – prognostic factors

		Events / Patients	Univariate			Multivariable (full) - 1			Multivariable reduced - 1		
			HR	95% CI	p-value	HR	95% CI	p-value	HR	95% CI	p-value
ER status (local)	Negative	7/62		1 (ref)			1 (ref)			--	
	Positive	18/120	1.35	0.56, 3.23	0.50	1.53	0.51, 4.63	0.45	-	-	-
PgR status (local)	Negative	13/101		1 (ref)			1 (ref)			--	
	Positive	12/81	1.06	0.48, 2.34	0.88	0.66	0.25, 1.77	0.41	-	-	-
Tumour grade	1-2	6/57		1 (ref)			1 (ref)			1 (ref)	
	3	16/83	2.33	0.93, 5.83	0.07	1.75	0.67, 4.56	0.25	1.67	0.66, 4.26	0.28
Tumour size	≤2cm	9/78		1 (ref)			1 (ref)			1 (ref)	
	>2cm	13/62	2.50	1.10, 5.67	0.028	2.18	0.93, 5.12	0.073	2.08	0.90, 4.78	0.085
Age	Continuous	25/182	0.98	0.94, 1.02	0.30	0.98	0.94, 1.02	0.24	-	-	-
FISH HER2 amplification Ratio	Continuous	25/182	0.96	0.88, 1.06	0.42	0.97	0.87, 1.07	0.45	-	-	-
Ki67 relative reduction at surgery*	<10% or none	7/55		1 (ref)			1 (ref)			1 (ref)	
	>50%	2/56	0.22	0.04, 1.07	LR test	0.22	0.04, 1.12	LR test	0.24	0.05, 1.21	LR test
	10-50%	16/64	1.95	0.79, 4.82	0.001	1.66	0.65, 4.24	0.005	1.82	0.73, 4.55	0.0039

*The differences in the Ki67 relative reduction are due to differences between >50% and 10-50% reduction, as observed in the KM figure 3A in the main manuscript.

8 Appendix 9 – Exploratory analysis of Tumour Lymphocytes Infiltrates (TILs)

Figure 8. Baseline TILs distribution by part and group

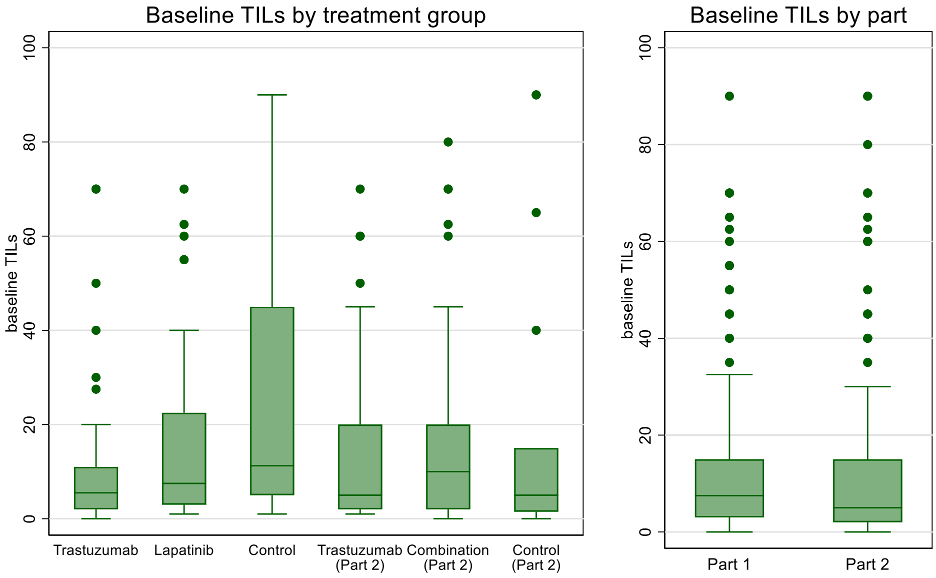

<u>Mann-Whitney test between treatment groups (Part 1):</u>

Lapatinib vs. Control: p = 0.21

Trastuzumab vs. Lapatinib: p = 0.19

<u>Mann-Whitney test between treatment groups (Part 2):</u>

Combination vs. Control: p = 0.20

Trastuzumab vs. Combination: p = 0.46

<u>Mann-Whitney test between groups:</u>

Part 1 vs. Part 2: p = 0.50

Figure 9. Logfold change in Ki67 by baseline TILs category over treatment

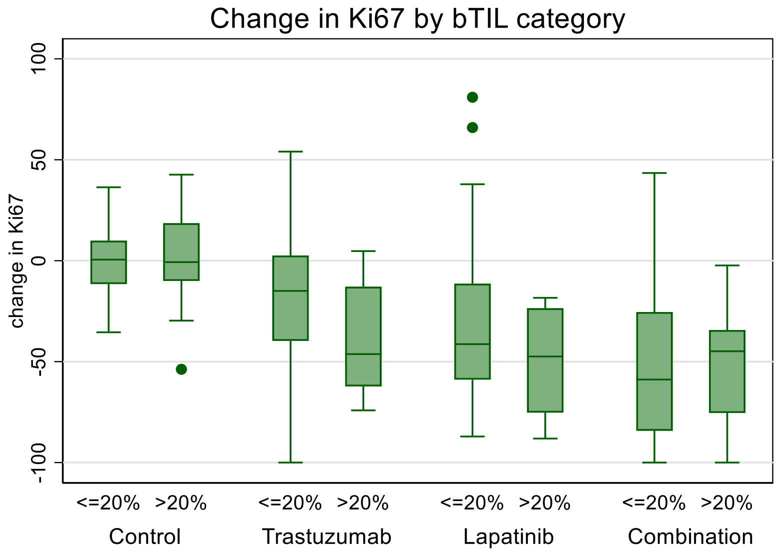

<u>Mann Whitney test between TILs groups (within treatment groups):</u>

Part 1+2 Trastuzumab: p = 0.04

Part 1 Lapatinib: p = 0.28

Part 1+2 Control: p = 0.80

Part 2 Combination: p = 0.96

Table 15. Change in TILs from baseline to surgery in patients with RCB2/3 tumours (n=191)

	Part 1&Part 2			
	**Trastuzumab**	**Lapatinib**	**Combination**	**Control**
**TILS**<br>N with paired data	69	43	33	46
Baseline TILS	5.5 (2-14)	7.5 (3,23.5)	10 (2,15)	5.1 (3,30)
Surgery TILS	10 (5-40)	12.5 (4,40)	15 (5,40)	7 (4,30)
Absolute change	4 (0-20)	2.5 (0,17.5)	4 (0,30)	0 (-1 , 5)
%Percentage change	91% (0-363)	39% (0,230)	55% (0,363)	0 (-20,65)
Log-fold change	0.65 (0-1.53)	0.32 (0,1.19)	0.44 (0,1.53)	0 (-0.22,0.50)
Kruskal-Wallis p-value:	0.0143			

It is worth noting that, as most pCR and RCB1 cases occurred in the combination group, and pCR and RBC1 are excluded from the TILs analysis, the combination group has less patients than other treatment groups.

Figure 10. Ki67 response by relevant change in TILs and treatment group in in patients with RCB2/3 tumours

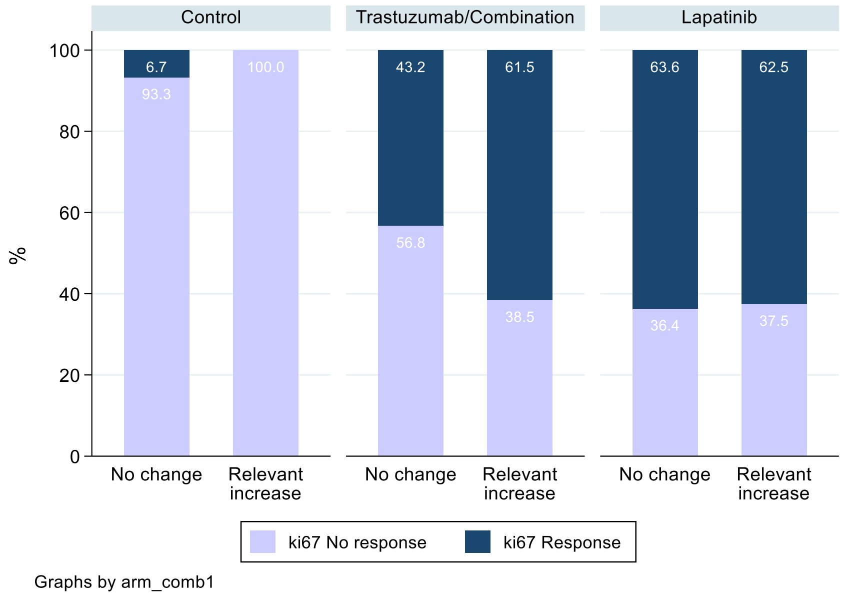

<u>Fisher test within treatment groups (p-value for relevant increase with Ki67 response):</u>
Control: 0.999
Trastuzumab & Combination: 0.118
Lapatinib: 0.999

9 Appendix 10 – Further safety details

Data on incidence of rash and diarrhoea during the peri-operative period was collected retrospectively for all trial patients. During pre-surgical treatment, rash (any grade) was reported in 61/117 (52%) patients who received lapatinib (alone or in combination) vs 1/89 (1%) in the trastuzumab only groups. Diarrhoea pre-surgery (any grade) was reported in 46/117 (39%) patients who received lapatinib vs 3/89 (3%) who received trastuzumab alone. No rash or diarrhoea episodes were reported in the control group. The table below provides details of all observed Serious Adverse Events.

Table 16. Serious Adverse Events observed during peri-operative treatment

#SAE	Group	Summary (CTC grade)	Type	Severity	Status	CI relatedness
1	Lapatinib	Raised ALT (2):	Other	2. Moderate	Recovered	4. Probable
2	Lapatinib	Diarrhoea(3): Stomach pain/cramps(2):	Hospitalization	2. Moderate	Recovered	4. Probable
3	Lapatinib	ALT > 3 ULN (2):	Other	1. Mild	Recovered	4. Probable
4	Lapatinib	Acneiform Rash to face/scalp/upper body (2):	Other	2. Moderate	Recovered	
5	Lapatinib	Diarrhoea(3): Post op hematoma of LD flap(3):	Prolongation of Hospitalization	2. Moderate	Recovered	4. Probable
6	Trastuzumab (p1)	In recovery from surgery, patient developed a hematoma, returned to theatre for evacuation + 2 units of blood transfusion(4):	Life Threatening	4. Life Threatening	Recovered	1. Unrelated
7	Trastuzumab (p1)	MI(4): Acute LVF on waking from GA(4):	Life Threatening	3. Severe	Recovered with Sequelae	3. Possible
8	Trastuzumab (p1)	Cellulitis to left breast(2)	Hospitalization	2. Moderate	Recovered	1. Unrelated
9	Trastuzumab (p1)	Headache, Dizzy, blurry vision(2):	Hospitalization	1. Mild	Recovered	3. Possible
10	Trastuzumab (p1)	Atrial Fibrillation(2):	Hospitalization	2. Moderate	Recovered with Sequelae	2. Unlikely
11	Trastuzumab (p2)	post-operative nausea/vomiting(2):	Prolongation of Hospitalization	2. Moderate	Recovered	1. Unrelated
12	Combination	Vomiting(3): Diarrhoea(3):	Hospitalization	3. Severe	Recovered	4. Probable
13	Combination	Non neutropenic sepsis with increased WBC. CRP, temperature cellulitis of surgical scar(3):	Hospitalization	3. Severe	Recovered with Sequelae	1. Unrelated
14	Combination	Acneiform facial rash very erythematous with pustules(3):	Other	3. Severe	Recovered	4. Probable
15	Combination	Hematoma(3):	Prolongation of Hospitalization	2. Moderate	Recovered	2. Unlikely
16	Combination	Abdominal Pain(4): Nausea and Vomiting(1): Loose Stools(1):	Other	3. Severe	Recovered	5. Definite
