# Supplementary figures and images for: Combined Perioperative Lapatinib and Trastuzumab in Early HER2-Positive Breast Cancer Identifies Early Responders: Randomized UK EPHOS-B Trial Long-Term Results
Source: Clin Cancer Res. 2022 Feb 14;28(7):1323–34. doi: 10.1158/1078-0432.CCR-21-3177 (PMC9610457; doi:10.1158/1078-0432.CCR-21-3177)

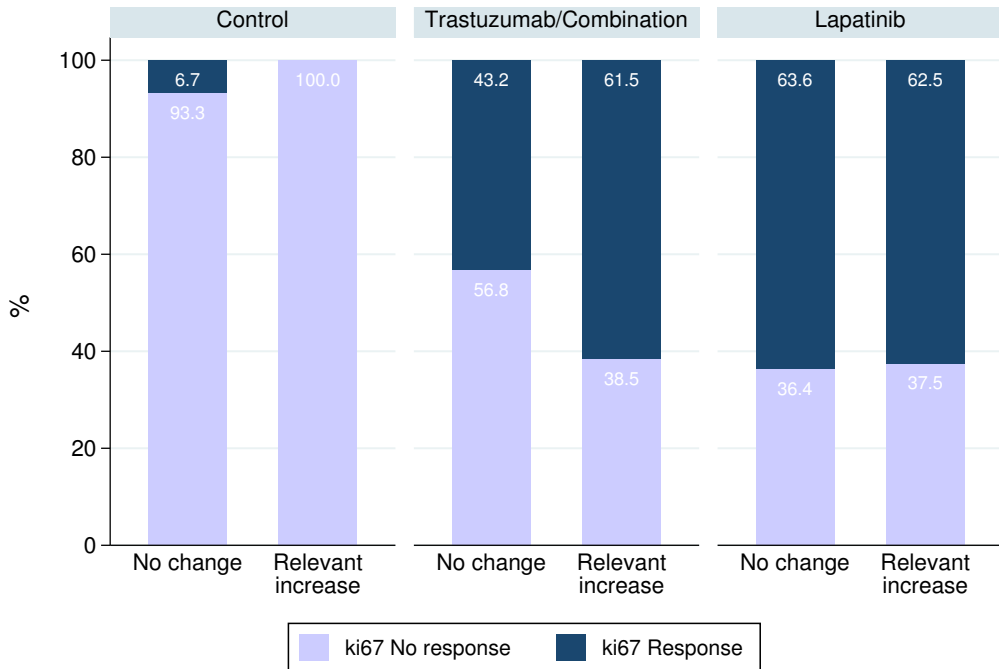

Graphs by arm\_comb1

Supplement: Supplementary Figure [file ccr-21-3177_supplementary_figure_10_supp10.pdf]

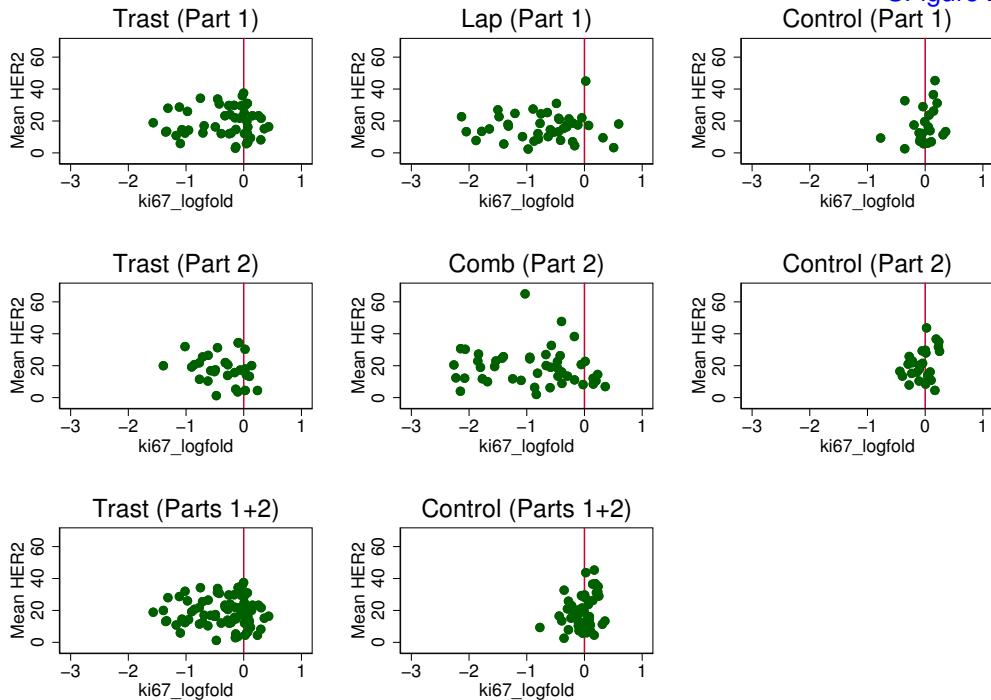

Supplement: Supplementary Figure [file ccr-21-3177_supplementary_figure_2_supp2.pdf]

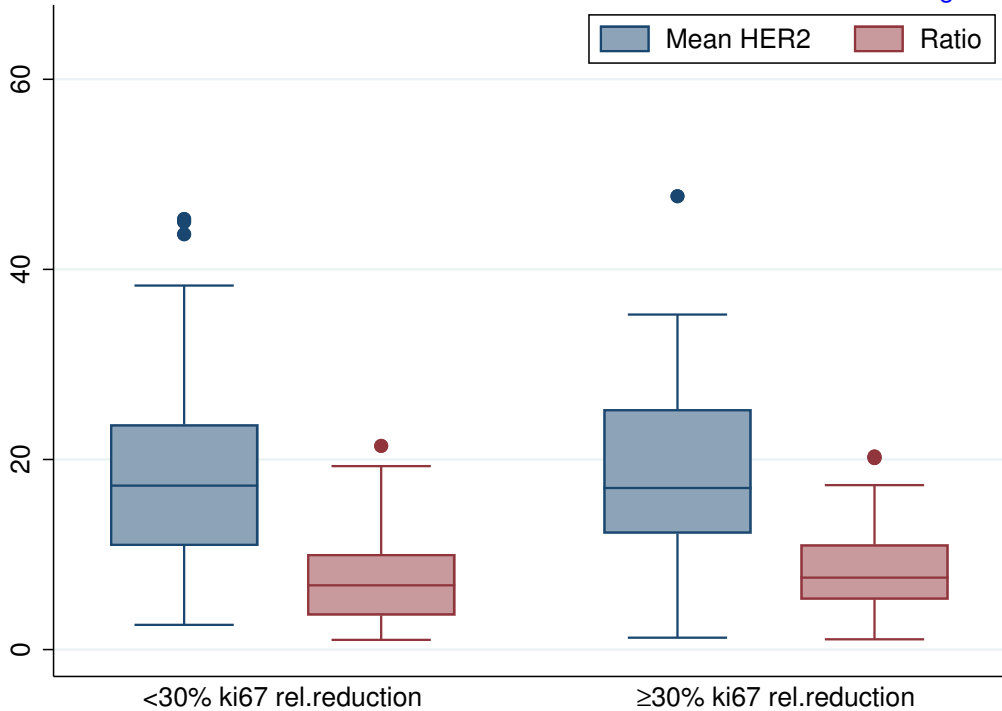

Supplement: Supplementary Figure [file ccr-21-3177_supplementary_figure_3_supp3.pdf]

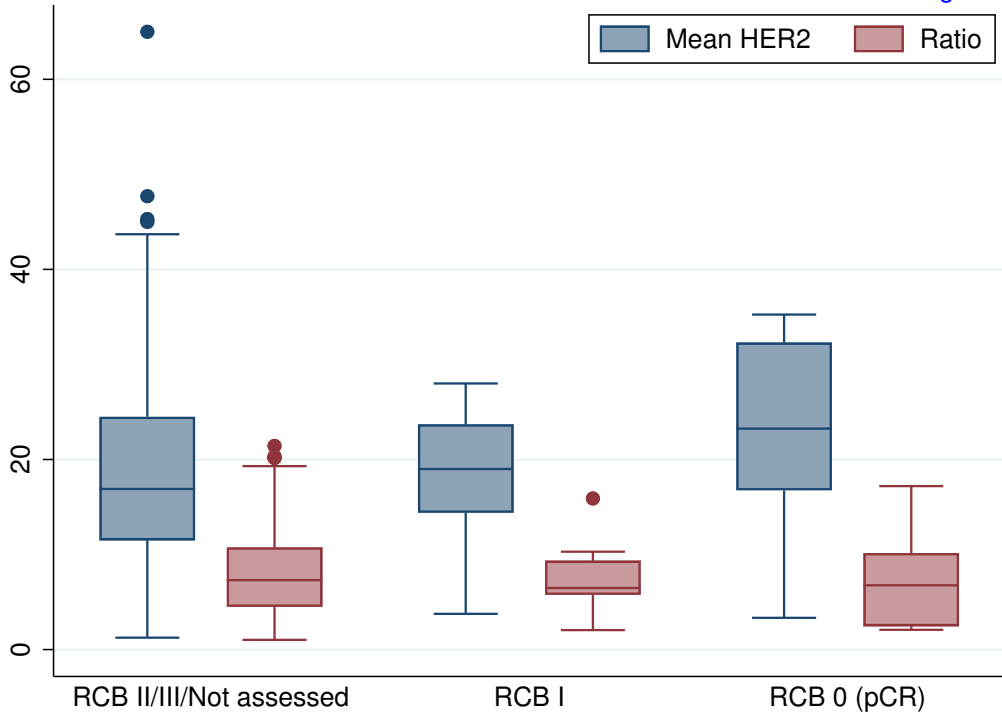

Supplement: Supplementary Figure [file ccr-21-3177_supplementary_figure_4_supp4.pdf]

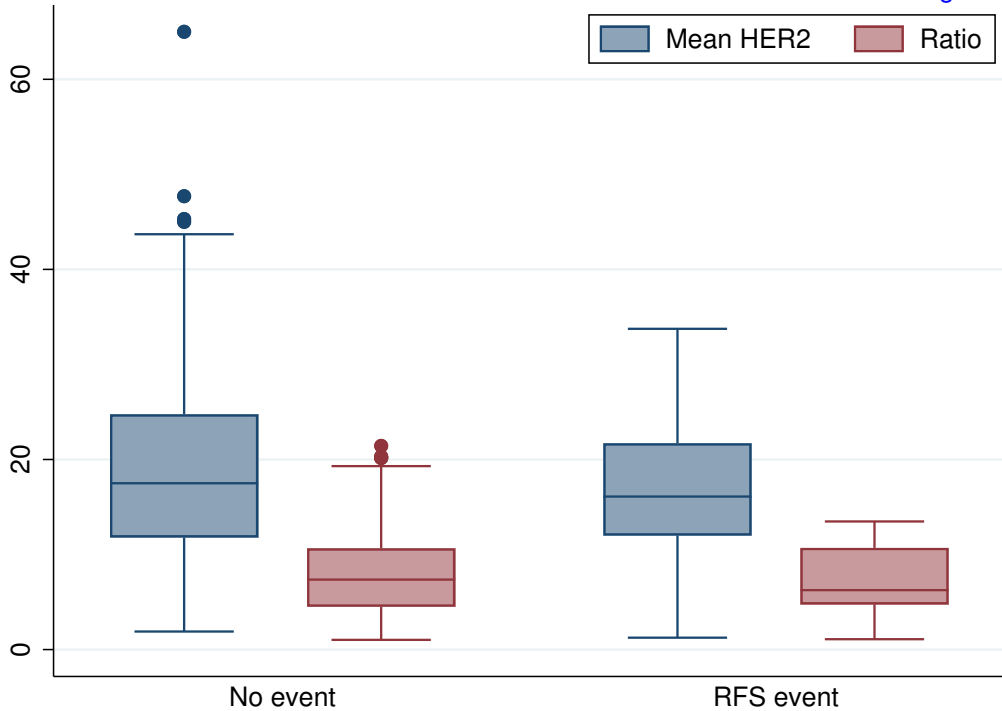

Supplement: Supplementary Figure [file ccr-21-3177_supplementary_figure_5_supp5.pdf]

## Baseline – all groups–

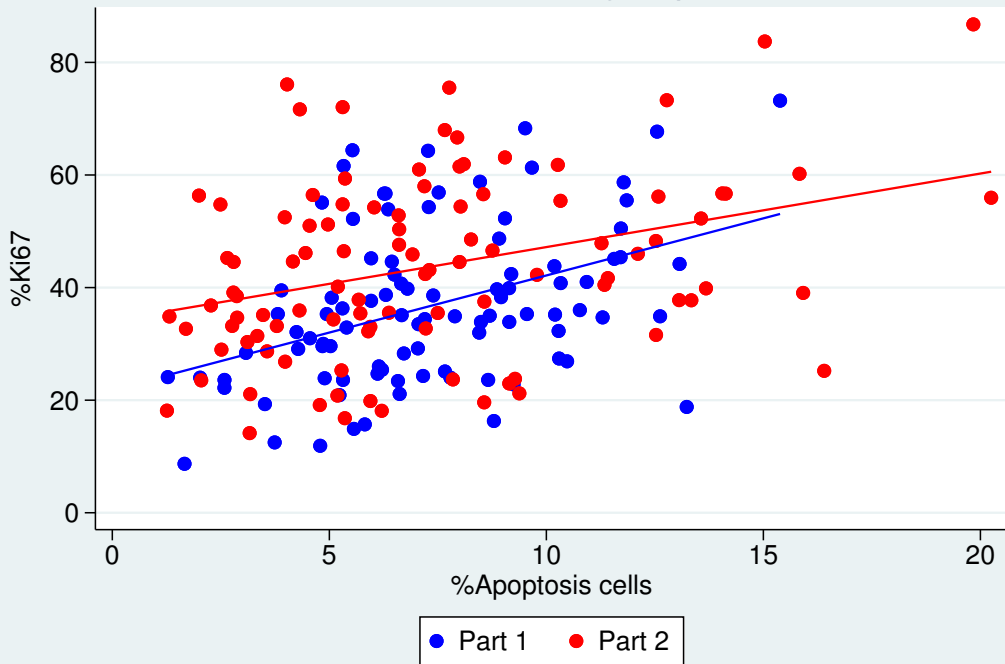

Supplement: Supplementary Figure [file ccr-21-3177_supplementary_figure_6_supp6.pdf]

## % change association

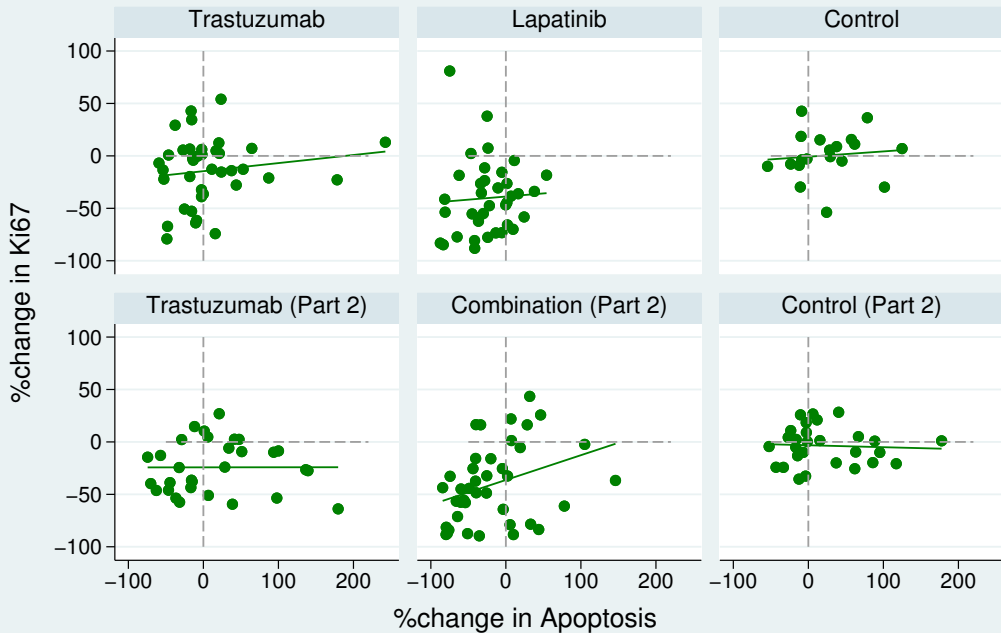

Graphs by Arm

Supplement: Supplementary Figure [file ccr-21-3177_supplementary_figure_7_supp7.pdf]

Baseline TILs by treatment group

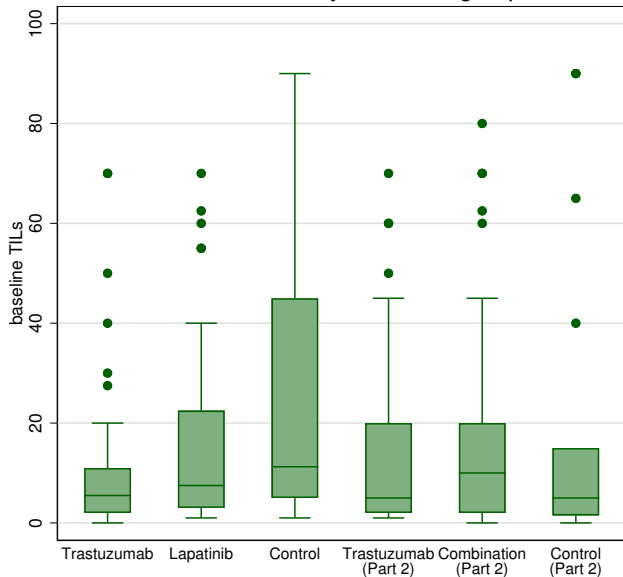

Baseline TILs by part

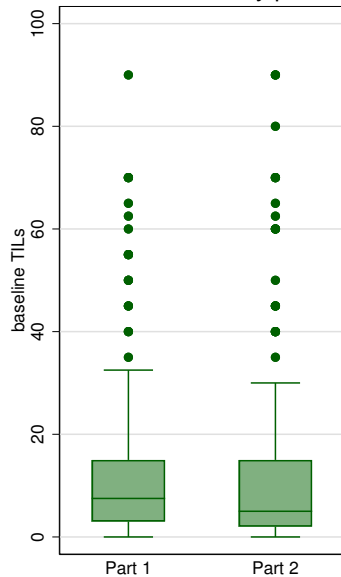

Supplement: Supplementary Figure [file ccr-21-3177_supplementary_figure_8_supp8.pdf]

## Change in Ki67 by bTIL category

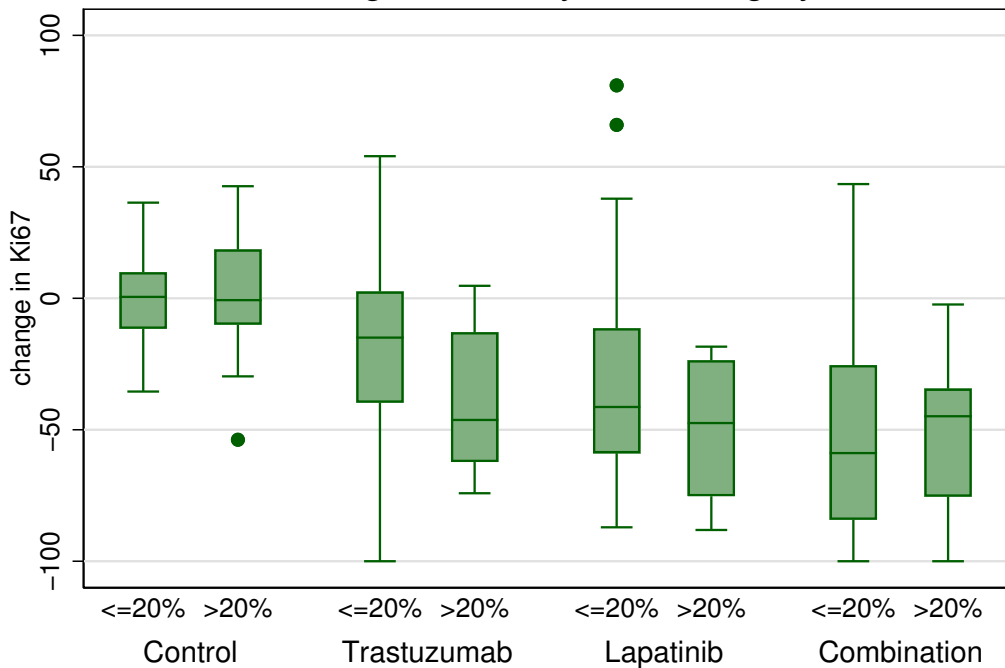

Supplement: Supplementary Figure [file ccr-21-3177_supplementary_figure_9_supp9.pdf]
