# Supplementary material for: Combined Perioperative Lapatinib and Trastuzumab in Early HER2-Positive Breast Cancer Identifies Early Responders: Randomized UK EPHOS-B Trial Long-Term Results
Source: Clin Cancer Res. 2022 Feb 14;28(7):1323–34. doi: 10.1158/1078-0432.CCR-21-3177 (PMC9610457; doi:10.1158/1078-0432.CCR-21-3177)
Supplement: Supplementary Figure [file ccr-21-3177_supplementary_figure_1_supp1.pdf]

SFigure 1

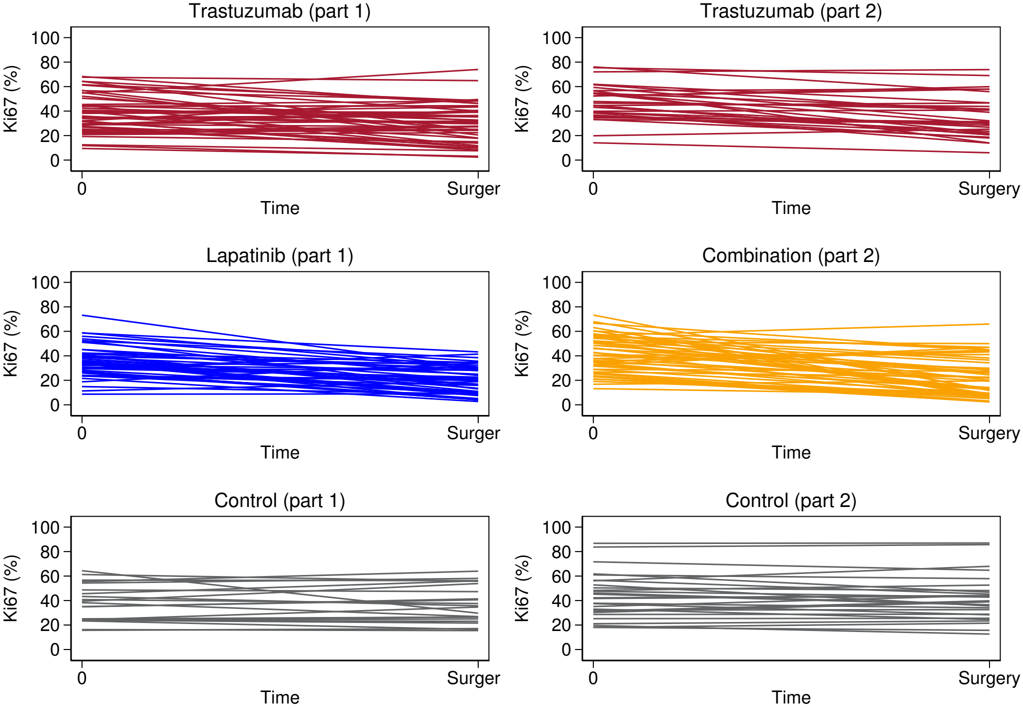
Trastuzumab (part 1)
Trastuzumab (part 2)
Lapatinib (part 1)
Combination (part 2)
Control (part 1)
Control (part 2)
Ki67 (%)
100
80
60
40
20
0
0
Surger
Surgery
Time
